# Tumor‐Intrinsic HEXB Drives Endothelial‐To‐Mesenchymal Transition and Confers Resistance to Anti‐Angiogenic Therapy in Glioblastoma

**DOI:** 10.1002/advs.78208

**Published:** 2026-10-08

**Authors:** Tianqi Liu, Xin Chen, Tao Sun, Jianqi Wu, Haixu Yan, Xing Liu, Hongda Lin, Xiangyu Wang, Ziao Xiao, Yang Xu, Qiang Fu, Peng Cheng, Wen Cheng, Anhua Wu

**Affiliations:** ^1^ Department of Neurosurgery Shengjing Hospital of China Medical University Shenyang Liaoning China; ^2^ Department of Neurosurgery The First Hospital of China Medical University Shenyang Liaoning China; ^3^ Department of Rehabilitation Shengjing Hospital of China Medical University Shenyang Liaoning China

**Keywords:** anti‐VEGF therapy, endothelial‐mesenchymal transition, glioblastoma, HEXB, HSP60, ubiquitination

## Abstract

Although anti‐angiogenic (anti‐VEGF) therapy is widely used for glioblastoma (GBM), its efficacy is severely limited by acquired resistance. Here, we identify endothelial‐mesenchymal transition (Endo‐MT) in brain endothelial cells as the primary driver of this resistance, fundamentally modulated by hexosaminidase B (HEXB). Mechanistically, HEXB directly binds and stabilizes heat shock protein 60 (HSP60) by inhibiting its ubiquitination and degradation. This stabilization upregulates insulin‐like growth factor binding protein 1 (IGFBP1), thereby promoting the Endo‐MT process. Addressing its clinical relevance, the combination of a HEXB inhibitor and anti‐VEGF therapy exerts potent synergistic therapeutic effects in GBM murine models. Overall, our study establishes HEXB as a key driver of Endo‐MT and provides a promising dual‐targeting strategy to overcome anti‐VEGF resistance in GBM.

## Graphical abstract

This study elucidates how glioblastoma tumors develop resistance to anti‐VEGF therapy through endothelial‐to‐mesenchymal transition (Endo‐MT). The researchers identify a novel signaling pathway where tumor‐derived HEXB stabilizes HSP60 to drive this vascular reprogramming. Pharmacological inhibition of HEXB successfully reverses Endo‐MT and re‐sensitizes tumors to treatment, proposing a new strategy to overcome therapeutic failure.

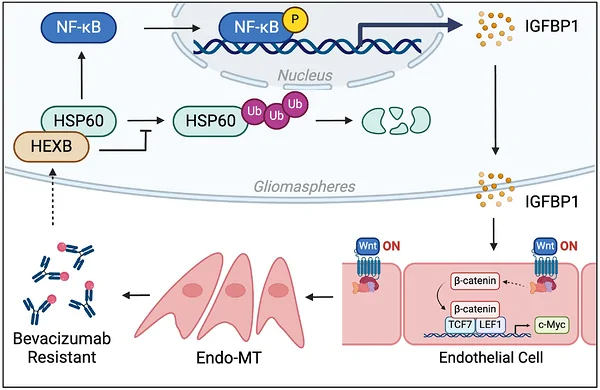

## 1 Introduction

Glioblastoma (GBM) is the most prevalent and aggressive primary malignant brain tumor, characterized by a highly infiltrative nature and robust microvascular proliferation [1]. Given its profound reliance on angiogenesis for survival and progression, targeting the tumor vasculature has been a major therapeutic focus. Consequently, anti‐angiogenic therapies, primarily utilizing the anti‐VEGF antibody bevacizumab, have been widely integrated into clinical practice [2, 3]. However, while anti‐VEGF treatment often yields temporary radiological improvements and reduces peritumoral edema, it ultimately fails to confer a significant overall survival benefit [4, 5]. Following initial vascular pruning, GBM invariably adapts to the therapeutic stress induced by VEGF blockade, deploying alternative, VEGF‐independent mechanisms to sustain tumor blood supply and facilitate aggressive recurrence.

Emerging evidence indicates that endothelial cell plasticity, specifically the endothelial‐mesenchymal transition (Endo‐MT), plays a pivotal role in driving this adaptive resistance [6]. During Endo‐MT, brain endothelial cells lose their canonical tight junctions and acquire a mesenchymal‐like phenotype, promoting vascular instability and resistance to anti‐angiogenic stress [7, 8]. Under the selective pressure of bevacizumab, the tumor microenvironment undergoes dynamic remodeling, wherein tumor cells secrete various paracrine factors to instruct this endothelial plasticity [9]. Despite the recognized clinical importance of Endo‐MT in bypassing VEGF dependency, the specific tumor‐intrinsic drivers and the precise molecular signaling networks that orchestrate this paracrine crosstalk during anti‐VEGF therapy remain incompletely elucidated. Identifying these actionable nodes is urgently needed to develop effective combination strategies for GBM.

In this study, we aimed to delineate the molecular underpinnings of anti‐VEGF therapeutic resistance and identify novel therapeutic vulnerabilities. We uncover hexosaminidase B (HEXB) as a previously unrecognized, anti‐VEGF‐inducible driver of paracrine Endo‐MT. Mechanistically, we demonstrate that HEXB directly interacts with heat shock protein 60 (HSP60), actively preventing its degradation. This aberrant stabilization of HSP60 triggers the persistent activation of the NFκB signaling pathway, culminating in the robust expression and secretion of insulin‐like growth factor binding protein 1 (IGFBP1). Crucially, utilizing preclinical murine models, we show that pharmacological inhibition of HEXB effectively reverses endothelial plasticity and restores sensitivity to VEGF blockade. Together, our findings define the HEXB/HSP60/IGFBP1 axis as a fundamental mechanism of tumor‐endothelial crosstalk and provide a strong rationale for targeting HEXB to overcome anti‐VEGF resistance in GBM.

## 2 Materials and Methods

### 2.1 Experimental Materials

All antibodies, reagents, software and primer sequencing used in this study were all listed in Table S1.

### 2.2 Gliomaspheres and Cell Line Culture

The gliomaspheres used in this study were generated from primary GBM tumors, and SB mGSCs were generated from a murine spontaneous GBM model previously reported [10, 11, 12]. Briefly, SB system contained transposon DNA and transposase, which could cut off the transposon DNA and paste it into the target genome. The transposon plasmid with genes (*Pdgfrb*/sh*Pten*/sh*Tp53*) and the plasmid encoding transposase were conducted respectively. These plasmids were mixed together and injected into the brain of fetal mouse (within 24 h after born) to induce spontaneous glioma mice model. The primary gliomaspheres (GSC1 and GSC21) and SB mGSCs were all cultured in DMEM/F‐12 medium (Gibco) containing 2% (vol/vol) B27 supplement (Gibco), epidermal growth factor (EGF, 20 ng/ml, Peprotech), basic fibroblast growth factor (bFGF, 20 ng/ml, Peprotech) and heparin (2.5 µg/ml, sigma). Only early passage GSC cells were used for the study. Human brain microvascular endothelial cells (HBMECs), HEK293 cells and U87 cells were maintained in Dulbecco's modified Eagle's medium (DMEM, 10566024, Gibco) supplemented with 10% fetal bovine serum (FBS, 16140071, Gibco) and 1% penicillin/streptomycin (10378016, Gibco). All cells mentioned above were cultured at 37°C with 5% CO_2_.

### 2.3 RNA Interference

IGFBP1‐targeting siRNAs (siIGFBP1), HSP60‐targeting siRNAs (siHSP60), ZNF311‐targeting siRNAs (siZNF311), ITGA5‐targeting siRNAs (siITGA5) and a negative control siRNA (siNC) synthesized by Sangon Biotech (Shanghai, China) were transfected into GSC1 and GSC21 cells with Lipofectamine 3000 reagent (L3000015, Invitrogen) according to the manufacturer's instructions. Before transfecting, the gliomaspheres were dissociated into single cells with Accutase and then seeded in six well plates coated with 0.5% laminin (L2020, Sigma) preparedly in advance. Cells were harvested for the next assay after incubated for 48 h.

### 2.4 Lentivirus Transfection

Lentiviruses carrying *HEXB* knockdown or overexpression were purchased from GeneChem, while the doxycycline (Dox)‐inducible *Hexb* knockdown lentivirus were purchased from OBiO Biotech. The lentivirus transduction was performed as the manufacturer's instructions. Before transfecting, the gliomaspheres were dissociated into single cells with Accutase and then seeded in six well plates coated with 0.5% laminin preparedly in advance. The transfected cells were then selected using 3 µg/ml puromycin for 15 days.

### 2.5 Plasmid Transfection

Plasmids harboring HEXB with Flag‐tag were purchased from GeneChem. Five truncated mutant plasmids (Δ80, Δ200, Δ350, Δ500 and Δ556) and one wildtype GST‐tagged plasmid for HEXB were purchased from OBiO Biotech. The plasmids carrying three peptides of HSP60 protein (1‐200aa, 201–400aa and 401–573aa) and one wildtype Myc‐tagged plasmid for HSP60 were purchased from OBiO Biotech. Plasmids harboring TRIM21 with Flag‐tag were purchased from OBiO Biotech. Plasmids harboring ubiquitin with HA‐tag were purchased from Sangon Biotech. Mutant ubiquitin plasmids (K6, K27, K29, K33, K48, K63, K6R, K29R and K63R) were also constructed from Sangon Biotech. Plasmid transfection was performed with Lipofectamine 3000 as directed by the manufacturer. Cells were harvested for the next assay after incubated for 48 h.

### 2.6 Conditional Medium Collection

For collecting the conditional medium, the gliomaspheres were dissociated into single cells with Accutase and then seeded in six well plates coated with 0.5% laminin preparedly. Then, the gliomaspheres were treated with different methods. After different treatments, the gliomaspheres were incubated with serum‐free medium and collected the supernatant after 24 h incubation. Supernatant was mixed with equal volume proportion of DMEM medium containing 10% FBS to prepare conditional medium (CM). For specific pharmacological or recombinant protein treatments, the indicated concentrations of Gal‐PUGNAc (2‐(AcetylaMino)‐2‐deoxy‐N‐[[(phenylaMino)carbonyl]oxy]‐D‐galactoniMidic Acid δ‐Lactone, 100 µM), rhHEXB (1 µg/ml) or rhIGFBP1 (1 µg/ml) were introduced into the CM to subsequently treat the HBMECs. HBMECs were harvested for the next assay after incubated with different CM for 24 h.

### 2.7 Co‐Culturing System

6‐well transwells (3450, Corning) were used for creating co‐culturing system. The gliomaspheres were dissociated into single cells with Accutase and then seeded in the upper chambers coated with 0.5% laminin preparedly in advance. After 24 h, the 6‐well transwell inserts with gliomaspheres were then transferred to another 6 well plate which was pre‐seeded with HBMECs and co‐cultured for another 48 h. The HBMECs in the lower chambers were harvested for the next assay.

### 2.8 Immunofluorescence Staining

HBMECs were treated with different CM for 24 h and then stained for immunofluorescence analysis. Cells were permeabilized with 0.5% Triton X‐100 (T8200, Solarbio) for 20 min. After 5% BSA incubation for 1 h, cells were incubated in VEGFR2, VE‐cadherin, N‐cadherin, S100A4, HEXB or HSP60 antibodies at 4°C overnight. Following incubation with fluorescein (FITC) or rhodamine (TRITC) secondary antibody and 4’,6diamidino‐2‐phenylindole (DAPI, C0060, Solarbio), the samples were detected using fluorescence microscope (Leica).

4 µm thick section slides from clinical GBM samples and murine brain tissues were washed three times in PBS. Then the sections were permeabilized with 0.5% Triton X‐100 for 20 min. After 5% BSA incubation for 1 h, sections were incubated in CD31, S100A4 and VEGFR2 antibodies at 4°C overnight. Following incubation with secondary antibody and 4’,6diamidino‐2‐phenylindole, the samples were detected using fluorescence microscope (Leica).

### 2.9 Cell Migration Assay

For detecting the migration ability of HBMECs, Transwell inserts with an 8 µm pore size (Corning, 3422) were used. The top chambers were filled with HBMECs with different treatments (5 × 10^4^ cells in 0.2 mL) in a medium containing 0.2% FBS. The lower chamber was supplemented with a medium containing 20% FBS. After incubation for 22–24 h, the chamber membrane was fixed using methanol and stained with a solution of 0.1% crystal violet. The migrated cells on the lower side of the membrane were observed and photographed using an upright microscope.

### 2.10 In Vitro Vascular Permeability Assay

For detecting the permeability of ECs, we refined the in vitro vascular permeability assay to validate the function change of endothelial cells. HBMECs with different treatments were seeded on Transwell inserts with 0.4 µm pore membrane in 6‐well plates, allowed to attach for 4 h, and cultured to reach confluence. FITC‐dextran (MW = 70,000 Da, Santa Cruz Biotechnology Inc., sc‐263323) was added to the top chamber. The medium from the lower chamber was collected, and fluorescence was measured with excitation wavelength at 485 nm and emission at 530 nm.

### 2.11 Western Blot

Total proteins from tissues and cells were extracted by whole cell lysis buffer (P0013B, Beyotime) and quantitated as described previously [13]. Same micrograms of protein from each sample were loaded onto a lane and electrophoresed using SDS‐PAGE followed by transfer to a polyvinylidene difluoride (PVDF) membrane (Millipore). After being blocked with 5% skimmed milk, the PVDF membranes were incubated with the primary antibody overnight at 4°C. Then, the PVDF membranes were incubated with the secondary antibodies at room temperature for 1 h. Protein expression was visualized with a chemiluminescence ECL reagent (180‐5001, Tanon).

### 2.12 Co‐immunoprecipitation

Total proteins from cells were lysed by cell lysis buffer (P0013, Beyotime) with PMSF (ST506, Beyotime). The lysates were first incubated with 50 µl Protein A/G for immunoprecipitation (B23202, Bimake) for 12 h at 4°C, and the samples were then incubated with appropriate antibodies (1 µg antibody per mg of cell lysates) for 2–3 h. The immunoprecipitated complexes were separated by SDS‐PAGE gel electrophoresis and followed by western blot experiments.

### 2.13 Real‐time Quantitative PCR (RT‐PCR)

Total RNA was isolated from cells as previously described [10]. Total RNA was reversely transcribed into cDNA with Prime‐Script RT Master Mix (TaKaRa). qPCR was performed with SYBR Green Master Mix (TaKaRa) for triplicate. The following conditions were used: 1 cycle of 95°C for 30s, followed by 40 cycles of a two‐step cycling program (95°C for 5s; 60°C for 30s). The mRNA expression of target genes was calculated by the 2 ^‐ΔΔCt^ method and normalized to 18s mRNA expression.

### 2.14 Enzyme‐Linked Immunosorbent Assay (ELISA)

The secretion of IGFBP1 was detected by ELISA following the manufacturer's instructions (Cloud‐Clone, SEA052Hu).

### 2.15 Flow Cytometry

The detailed protocol of flow cytometry was done as previously described [10]. FACS data were analyzed using FlowJo software (version 10.4).

### 2.16 Single‐Cell RNA (ScRNA) Sequencing

The tumor samples were obtained from SB‐bearing mice model under different treatment and prepared single‐cell suspensions. The CD31 Microbeads (130‐097‐418, Miltenyi) were used for positive selection CD31^+^ endothelial cells from single‐cell suspensions of mouse tissues. Single cell RNA seq libraries were prepared using 10× Genomics reagents and instruments. Library quality was verified using a Qubit 4.0 fluorometer and an Agilent 2100 Bioanalyzer. Sequencing was performed on the Illumina NovaSeq 6000 platform at a depth of ≥50,000 reads per cell with 150 bp paired end reads (performed by Biomarker Technologies Corporation, Beijing, China). Raw sequencing reads were aligned to the reference genome using CellRanger software; high‐quality cells expressing >500 genes and with a mitochondrial gene ratio <0.20 were retained, yielding a total of 36,000 qualified cells for downstream analysis. Samples were integrated using the IntegrateData function in Seurat with dimensions 1–20 to remove batch effects, followed by principal component analysis (PCA), and the top 30 principal components were used for unsupervised clustering (resolution = 0.5), UMAP, and t‐SNE visualization. Cell‐type signature scores were calculated using AddModuleScore with 100 control genes; endothelial and mesenchymal signatures were defined by Kdr/Cdh5 and Acta2/Aifm2, respectively. DEGs between groups were identified using FindMarkers (adjusted *p* < 0.05, log2FC < −1), and Gene Ontology (GO) enrichment analysis was performed using clusterProfiler with the org.Mm.eg.db database, with significant terms visualized as bar plots.

### 2.17 Single‐nuclei RNA (SnRNA) Sequencing

GBM specimens were minced in pre chilled lysis buffer, gently homogenized, and incubated on ice. Lysis was terminated by adding pre chilled 2% BSA solution. The mixture was filtered and centrifuged to collect nuclear pellets. Pellets were resuspended, stained with propidium iodide (PI), and purified using a BD FACSAria II flow cytometer to remove debris. Pure single nucleus suspensions were loaded onto a 10× Genomics chip to target ∼8,000 nuclei. cDNA amplification and library preparation were performed following the manufacturer's protocol. Sequencing was carried out on the Illumina NovaSeq 6000 platform by LC Bio Technology (Hangzhou, China) at ≥20,000 reads per nucleus with 150 bp paired end reads. Sequencing data processing, quality control, data integration, dimensionality reduction, clustering, cell type signature scoring, differential gene expression analysis were performed using the same computational pipelines and parameters described in Section 2.16.

### 2.18 Intracranial Mouse Model

Four‐to‐six‐week‐old male C57BL6 mice and BALB/c nude mice purchased from Beijing Vital River Laboratory Animal Technology were used to establish intracranial GBM xenografts. All mice were resided in specific pathogen‐free (SPF) conditions.

For analyzing the effect of VEGF‐A antibody on GBM progression in vivo, a sum of 5×10^4^ SB mGSCs were intracranially injected into the C57BL6 mice brains. After random grouping, mice were divided into two group: control (treated with IgG isotype control antibody) and VEGF‐A mAb group (treated with VEGF‐A antibody). VEGF‐A antibody was intraperitoneally injected at day 7 and 14 at a dose of 100 µg/mouse. SB‐bearing mice were sacrificed at day 11, 14, 17 for further H&E staining or were sacrificed at day 19 for further analysis or observed until death for survival analysis.

For analyzing the in vivo effects of HEXB on Endo‐MT process, 3 µl cell suspension (shNC/shHEXB: 2×10^5^) were injected into the BALB/c nude mice brains. Mice were sacrificed at same time for further flow cytometry analysis.

For analyzing the in vivo effects of HEXB inhibitor (Gal‐PUGNAc) on GBM progression and Endo‐MT process, a sum of 5×10^4^ SB cells were intracranially injected into the C57BL6 mice brains or a sum of 5×10^5^ U87 cells were intracranially injected into the BALB/c nude mice brains. After random grouping, mice were divided into two group: control (treated with saline as control) and Gal‐P group (treated with Gal‐PUGNAc). Gal‐PUGNAc was administrated intravenously (5 mg/kg) every two days after GBM cells were inserted for 5 days. SB‐bearing mice were sacrificed at day 16 for further analysis or observed until death for survival analysis. U87‐bearing mice were sacrificed at day 28 for further analysis or observed until death for survival analysis.

For analyzing the effect of HEXB inhibitor combined with anti‐VEGF therapy, a sum of 5×10^4^ SB cells were intracranially injected into each mouse. After random grouping, mice were divided into four group: control (treated with IgG isotype control antibody), Gal‐P group (treated with Gal‐PUGNAc alone), VEGF‐A mAb group (treated with VEGF‐A antibody alone) and combine group (treated with Gal‐PUGNAc and VEGF‐A antibody). VEGF‐A antibody was intraperitoneally injected at day 7 and 14 at a dose of 100 µg/mouse. Gal‐PUGNAc was administrated intravenously (5 mg/kg) every two days after GBM cells were inserted for 5 days. SB‐bearing mice were sacrificed at day 16 for further analysis or observed until death for survival analysis.

For analyzing the effect of HEXB depletion combined with anti‐VEGF therapy, SB cells were priorly transduced with a doxycycline (Dox)‐inducible *Hexb* knockdown lentivirus, and the knockdown efficiency upon Dox induction was validated. A sum of 5×10^4^ SB cells were intracranially injected into each mouse. After random grouping, mice were divided into four group: control (treated with IgG isotype control antibody), Dox group (treated with Dox alone), VEGF‐A mAb group (treated with VEGF‐A antibody alone) and combine group (treated with Dox and VEGF‐A antibody). VEGF‐A antibody was intraperitoneally injected at day 7 and 14 at a dose of 100 µg/mouse. 2 mg/mL of Dox was added into drinking water after GBM cells were inserted for 5 days. Drinking water was replaced every two days. SB‐bearing mice were sacrificed at day 16 for further analysis or observed until death for survival analysis.

### 2.19 Statistical Analysis

GraphPad Prism 10, and R 4.0.2 (https://www.r‐project.org/) software were used for statistical analysis. The prognostic difference was evaluated by Kaplan‐Meier survival analysis and log‐rank test. Pathway activity was inferred via ssGSEA using the R package GSVA. Preprocessed RNA‐seq data was analyzed against well‐recognized gene sets. ssGSEA scores were calculated using the Kolmogorov‐Smirnov test statistic with 1000 random permutations, converted to normalized enrichment scores (NES) for technical correction, and z‐score standardized across all samples to enable direct inter‐group comparison. Date were presented as mean ± standard deviation. Student's t test was used to assess differences and statistical significance was defined as a 2‐tailed *p* value < 0.05.

### 2.20 Study Approval

All experimental protocol was approved by the Ethics Committee of Shengjing Hospital of China Medical University. Animal experiments were conducted in accordance with the China Medical University Animal Care and Use Committee guidelines and approved by the Institutional Review Board of Shengjing Hospital of China Medical University.

## 3 Results

### 3.1 The Endo‐MT Process Contributed to the Failure of Anti‐VEGF Therapy in a GBM Mice Model

To explore the therapeutic efficacy of anti‐VEGF therapy in GBM, we established a spontaneous GBM mice model using the sleeping beauty (SB) system, then treated the mice with saline or a VEGF‐A antibody (Figure 1A). The mice did not display significantly prolonged survival following the VEGF‐A antibody treatment, demonstrating that our spontaneous GBM mice model was resistant to anti‐VEGF therapy (Figure 1B). We further collected the mice brain tissues on different days post‐implantation to monitor the tumor growth by H&E staining, which indicated that the tumor area grew steadily over time in the control group. In the VEGF‐A antibody group, the tumors were well‐controlled in the early treatment stage, but grew rapidly as the treatment progressed (Figure 1C and Figure S1A). Because the anti‐VEGF therapy primarily targeted ECs in GBM, we hypothesized that these cells had certain alterations during the anti‐VEGF therapy period that contributed to the rapid deterioration. Therefore, we extracted all cells from the tumors and performed magnet‐activated CD31^+^ cell sorting to enrich the ECs. ScRNA sequencing was further performed to detect the different states of CD31^+^ ECs following VEGF‐A antibody treatment (Figure 1D and E). Functional enrichment revealed that pathways correlating with EC plasticity were up‐regulated in the VEGF‐A antibody group, such as the establishment or maintenance of cell polarity, positive regulation of cell morphogenesis, and positive regulation of cytoskeleton organization (Figure 1F). These results suggested that the anti‐VEGF therapy possibly induced the Endo‐MT process of ECs in GBM. Furthermore, 19 cell populations were acquired by tSNE analysis (Figure S1B). We used a well‐recognized gene list to reflect the different Endo‐MT statuses of the ECs. Cluster 11 was regarded as endothelial‐like ECs, while clusters 0,1, and 7 were defined as mesenchymal‐like ECs (Figure S1C). Almost all (96.5%) of the endothelial‐like ECs appeared in the control group, while 89% of the mesenchymal‐like ECs present in the VEGF‐A antibody group (Figure 1G). Similar results were found with respect to treatment. 67.4% of the ECs in the VEGF‐A antibody group were mesenchymal‐like ECs, while this proportion was only 8.5% in the control group (Figure 1G). We also performed pseudotime analysis to reflect the dynamic changes in the ECs following VEGF‐A antibody treatment. The results illuminated that the endothelial‐like ECs transformed into mesenchymal‐like ECs after VEGF‐A antibody treatment (Figure S1D–H). Three‐color immunofluorescence staining was further performed to detect the Endo‐MT status of the ECs with different treatments. Mesenchymal‐like ECs (CD31^+^S100A4^+^ cells) were enriched in the VEGF‐A antibody group, while endothelial‐like ECs (CD31^+^VEGFR2^+^ cells) disappeared following VEGF‐A antibody treatment (Figure 1H). Overall, these findings confirmed that anti‐VEGF treatment could induce the Endo‐MT process of ECs, which led to GBM progression and anti‐VEGF therapeutic resistance.

**FIGURE 1:**
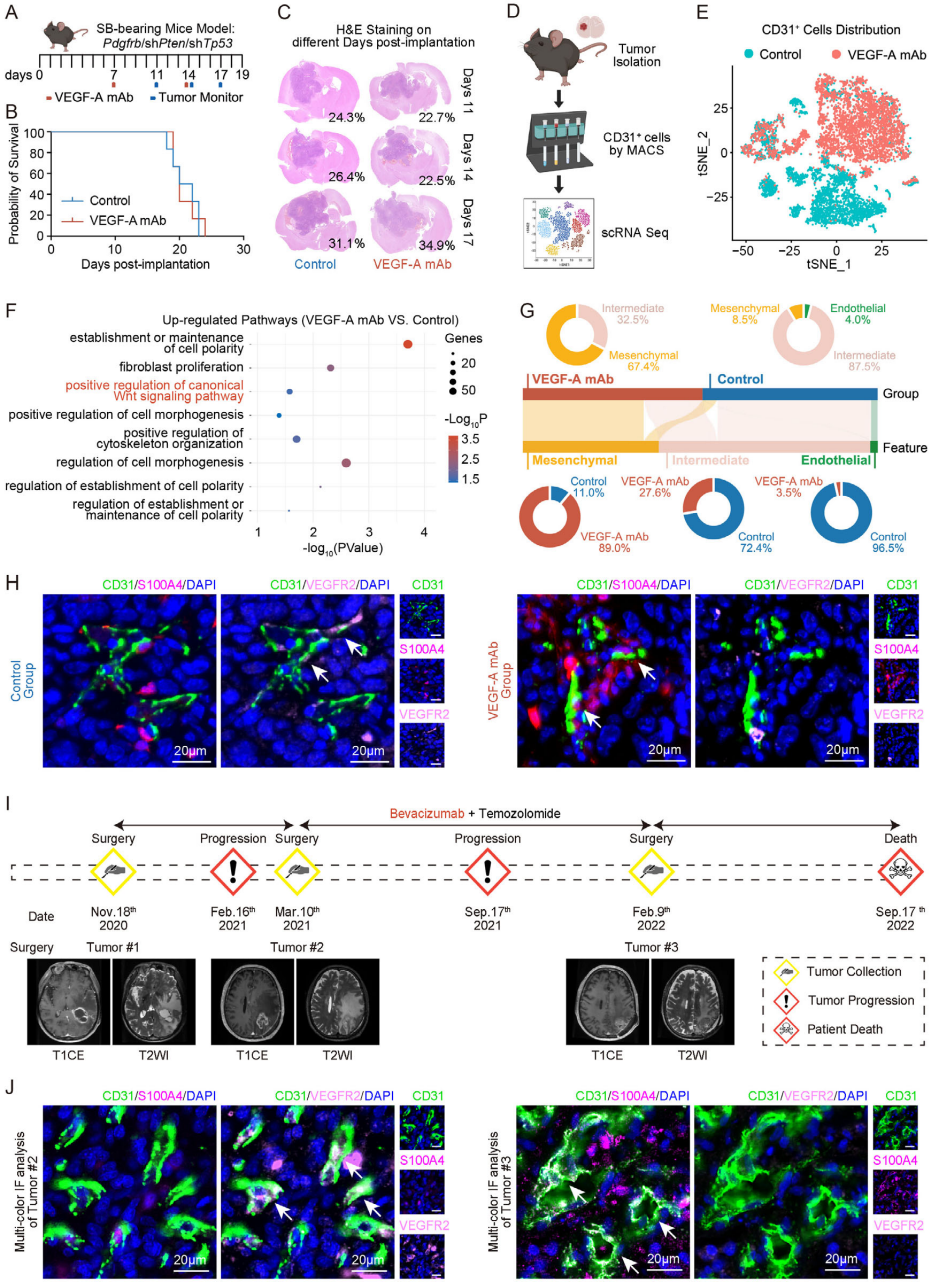
Endo‐MT process referred to the failure of anti‐VEGF treatment. (A) Schematic illustration of VEGF‐A antibody treatment in SB‐bearing C57BL6 mice. (B) Survival of SB‐bearing C57BL6 mice under different treatments (log‐rank test, n = 6). (C) The images of H&E staining of tumors from SB‐bearing C57BL6 mice under different treatments (numbers means the ratio of tumor area in whole brain). (D) Schematic illustration of scRNA sequencing analysis of tumors from SB‐bearing mice under different treatments. (E) t‐SNE plot of single‐cell RNA sequencing data in control group and VEGF‐A antibody treatment group. (F) Pathway enrichment analysis showing up‐regulated pathways in VEGF‐A antibody treatment group compared with control group. (G) The proportion of different subpopulations in different group. (H) Three‐color immunofluorescence staining image of tumors in control group and VEGF‐A antibody treatment group. (I) Schematic illustration of one GBM patient received Bevacizumab treatment. (J) Three‐color immunofluorescence staining image of different clinical GBM samples.

### 3.2 The Effect of Anti‐VEGF Therapy on Inducing Endo‐MT in ECs was Examined in a GBM Patient

To verify these findings in GBM patients, we examined samples from a clinical GBM patient who received bevacizumab therapy. This patient was first diagnosed with GBM and received the first surgery on 18 November, 2020. However, the tumor recurred and the patient received a second surgery on 10 March, 2021, after which bevacizumab treatment was administered. Unfortunately, tumor recurrence occurred again and a third surgery was performed on 9 February, 2022 (Figure 1I). Considering tumor progression, we collected tumor samples from the second surgery (Tumor #2) and third surgery (Tumor #3) for further examination (Figure 1I). Because there was still tumor recurrence with bevacizumab treatment between Tumor #2 and #3, this patient was defined as resistant to anti‐VEGF therapy. The response to anti‐VEGF therapy of this case was similar to the spontaneous GBM mice model we established (Figure 1A–C). Three‐color immunofluorescence staining confirmed an enrichment of mesenchymal‐like ECs (CD31^+^S100A4^+^ cells) following bevacizumab treatment, while the endothelial‐like ECs (CD31^+^VEGFR2^+^ cells) disappeared with the treatment (Figure 1J and Figure S1I). These results suggested that anti‐VEGF therapy could accelerate the EC Endo‐MT changes, leading to the malignant progression and development of anti‐VEGF therapy resistance in GBM.

### 3.3 Glioma‐Endogenous HEXB was Closely Related to Endo‐MT Changes

To examine the effects of anti‐VEGF treatment on Endo‐MT changes in vitro, we cultured human brain microvascular endothelial cells (HBMECs) with different doses of bevacizumab (Figure 2A). The HBMECs treated with bevacizumab showed no significant changes in endothelial markers (VEGFR2 and VE‐cadherin) or mesenchymal markers (N‐cadherin and S100A4) expression patterns (Figure 2B and Figure S2A). Western blot analysis also indicated that bevacizumab treatment did not affect the Endo‐MT status of the HBMECs (Figure 2C). However, when the HBMECs were co‐cultured with GSC1/GSC21 cells, both primary gliomaspheres derived from GBM specimens, bevacizumab treatment could diminish endothelial marker expression in the HBMECs. Additionally, mesenchymal marker expression was increased following bevacizumab treatment (Figure 2D,E and Figure S2B,C). These results suggested that anti‐VEGF therapy led to the Endo‐MT process by the manipulation of glioma cells on ECs.

**FIGURE 2:**
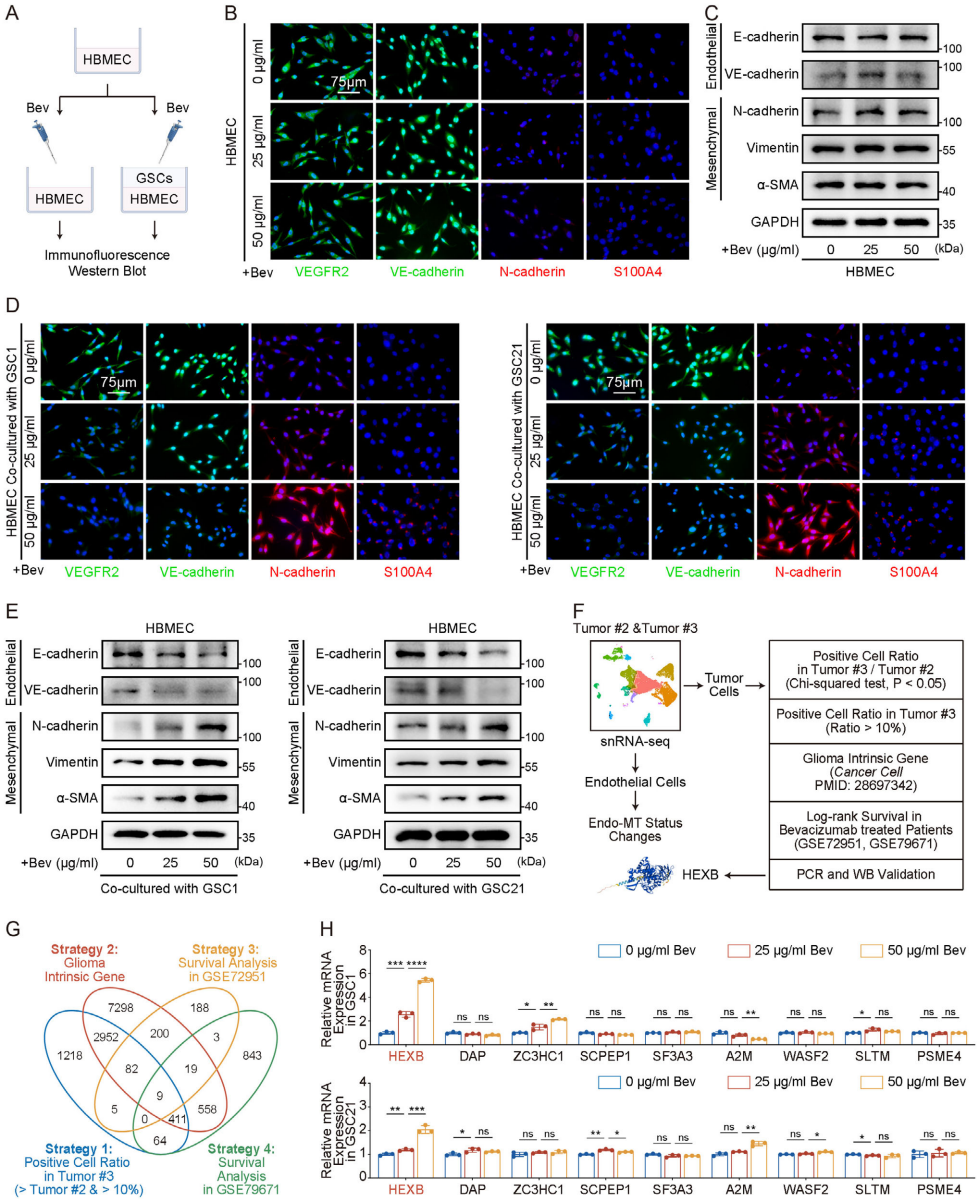
Identifying crucial gene involved in Endo‐MT process and the failure of bevacizumab based on clinical GBM samples. (A) Schematic illustration of the experiments under different conditions. (B) Immunofluorescence staining image of HBMECs treated with bevacizumab directly. (C) Western blot results of HBMECs treated with bevacizumab directly. (D) Immunofluorescence staining image of HBMECs under different treatments. (E) Western blot results of HBMECs under different treatments. (F) Work flow of the screening strategy. (G) The screening strategies of identifying crucial genes involved in Endo‐MT process and the failure of bevacizumab. (H) PCR results of GSC1 and GSC21 under different treatments. (Date were presented as means ± standard deviations, ns indicated *p* > 0.05, * indicates *p* ≤ 0.05, ** indicates *p* < 0.01, *** indicates *p* < 0.001, **** indicates *p* < 0.0001).

Thus, we aimed to determine the specific genes expressed in the tumor cells that was related to the Endo‐MT changes (Figure 2F). SnRNA sequencing was performed on clinical GBM tumor samples in Figure 1I (Figure S3). The proportion of N‐cadherin^+^ cells in ECs significantly increased following bevacizumab treatment, which was consistent with the immunofluorescence data showing that bevacizumab treatment aggravated the Endo‐MT changes in ECs (Figure S4). The tumor cells were then collected for further analysis using the snRNA sequencing data of Tumor #2 and #3 (Table S2). Given that the recurrent tumor (Tumor #3) emerged following Bevacizumab therapy and exhibited markedly enhanced Endo‐MT activity compared to Tumor #2, this case represents a clear instance of acquired anti‐VEGF resistance. To identify the active drivers of this resistant phenotype, we specifically focused on gain‐of‐function alterations. Firstly, we examined the percentage of which gene positive cells were upregulated following bevacizumab treatment, while the percentage of these cells were over 10% in Tumor #3. The results showed that 4741 genes had a positive cell ratio above 10%, with increased following bevacizumab treatment (Figure 2G, Screening Strategy 1). Secondly, because of the glioma‐endogenous role of bevacizumab on EC Endo‐MT changes, a glioma intrinsic gene list was intersected (Figure 2G, Screening Strategy 2). Thirdly, we analyzed two clinical datasets (GSE72951 and GSE79671), which include the prognostic information and gene expression data of GBM patients who received bevacizumab therapy. Genes that showed a strong correlation with poor prognosis by the Log‐rank test were then intersected in GSE72951 (Figure 2G, Screening Strategy 3) and GSE79671 (Figure 2G, Screening Strategy 4). After applying these filtering strategies, nine candidate genes met the criteria (Figure 2G). Finally, after treating GSC1 and GSC21 cells with different doses of bevacizumab, only HEXB expression levels were significantly boosted in a dose‐dependent manner (Figure 2H). Therefore, we selected HEXB as the main gene of interest.

Having established that bevacizumab treatment elevated HEXB expression in GSCs (Figure S5A and B), we next sought to delineate the upstream molecular mechanisms governing this phenomenon. Recognizing that in vitro blockade of autocrine VEGF signaling exerts a targeted stress on glioma cells, we hypothesized that this upregulation is driven by a specific stress‐responsive transcription factor. To identify this regulator, we performed RNA sequencing (RNA‐seq) on bevacizumab‐treated GSCs to profile treatment‐induced transcriptomic alterations. Concurrently, we interrogated the ChIP‐Atlas database to predict putative transcription factors harboring direct binding motifs within the *HEXB* promoter. Intersection of the significantly upregulated genes from our RNA‐seq dataset with the predicted HEXB transcriptional regulators prominently yielded ZNF311 as the primary upstream candidate (Figure S5C). Western blot analyses confirmed that bevacizumab upregulated both ZNF311 and HEXB expression (Figure S5A). We subsequently silenced ZNF311 expression via small interfering RNA, which attenuated the expression of HEXB (Figure S5D). To ascertain whether this transactivation was direct, dual‐luciferase reporter assays were conducted, which explicitly demonstrated that ZNF311 interacted with the HEXB promoter region to drive its transcriptional activity (Figure S5E). Crucially, to definitively determine whether ZNF311 is indispensable for the bevacizumab‐mediated HEXB induction, we detected that depletion of ZNF311 effectively abrogated the bevacizumab‐induced upregulation of HEXB (Figure S5F). Collectively, these findings provide a ZNF311‐orchestrated transcriptional reprogramming event deployed by GSCs to adapt to anti‐VEGF therapeutic stress.

### 3.4 HEXB Promoted Endo‐MT Changes

To further examine the relationship between HEXB and endothelial cells, we analyzed the expression of HEXB and CD31 (endothelial cell marker) in our in‐house cohort. There was a significant correlation between the levels of HEXB and CD31 (Figure S6A). Samples with high HEXB expression had increased expression of CD31, while high CD31 samples also owned more HEXB expression (Figure S6B and C). We also compared the expression of HEXB among different samples from each patient. There was a significant difference of HEXB expression between samples with highest CD31 expression and lowest CD31 expression in each patient (Figure S6D). Moreover, HEXB was evaluated in “microvascular proliferation” region than other regions in Ivy Atlas Project (Figure S6E). Significant correlations between HEXB and CD31 was confirmed in CGGA, TCGA and GSE16011 database (Figure S6F), while GBM patients with high HEXB expression possessed higher CD31 expression than GBM patients with low HEXB expression (Figure S6G). Immunohistochemistry staining of HEXB and CD31 was also conducted. The CD31 expression in tumor tissues with high HEXB levels was significantly increased compared with that in tumor tissues with low HEXB expression (Figure S6H and I). Compared to GBM patients with low HEXB expression, those with high HEXB expression were associated with a worse prognosis and a decreased therapeutic response rate under bevacizumab treatment (Figure 3A–C). These results demonstrated that HEXB was correlated with EC phenotype changes, which led to bevacizumab therapeutic failure.

**FIGURE 3:**
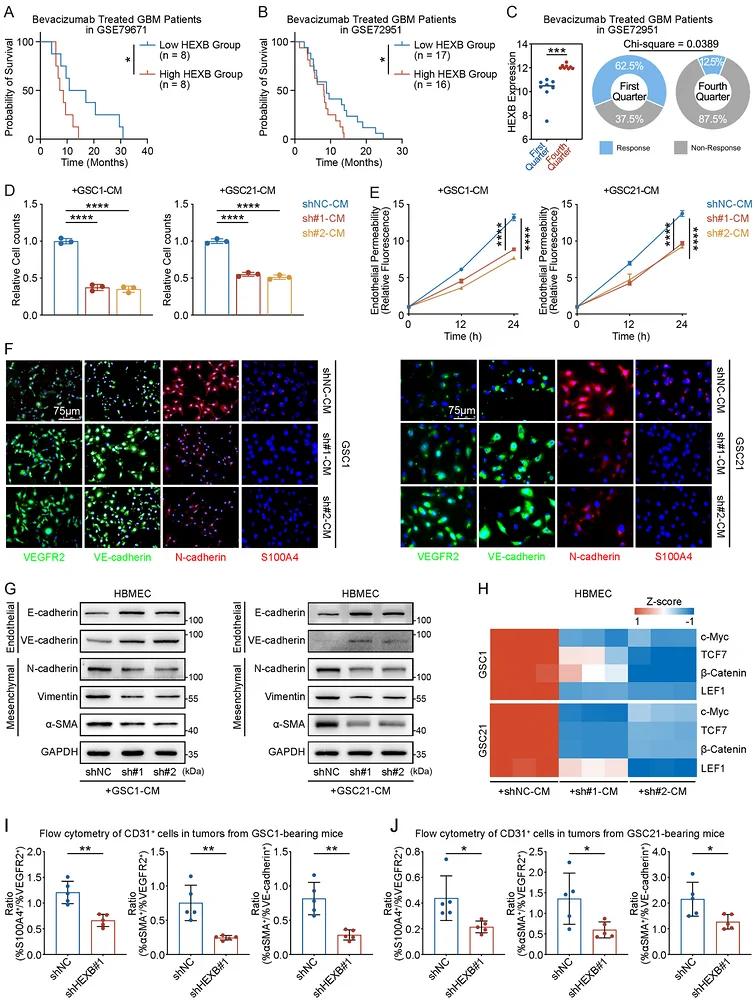
Glioma‐intrinsic HEXB promoted Endo‐MT changes of ECs. (A) Survival of GBM patients received bevacizumab treatment with different HEXB expression in GSE79671 (log‐rank test). (B) Survival of GBM patients received bevacizumab treatment with different HEXB expression in GSE72951 (log‐rank test). (C) The rate of response patients was lower in GBM patients with high HEXB expression in GSE72951 (Patients with a survival time superior to the median survival of the standard treatment were defined as the responder group, whereas those with a shorter survival time were defined as the non‐responder group). (D) The analysis of migration ability of HBMECs treated with different CM (sh#1: shHEXB#1; sh#2: shHEXB#2). (E) Permeability changes of HBMECs treated with different CM (sh#1: shHEXB#1; sh#2: shHEXB#2). (F) Immunofluorescence staining image of HBMECs treated with different CM (sh#1: shHEXB#1; sh#2: shHEXB#2). (G) Western blot results of HBMECs treated with different CM (sh#1: shHEXB#1; sh#2: shHEXB#2). (H) PCR results of HBMECs treated with different CM (sh#1: shHEXB#1; sh#2: shHEXB#2). (I) Flow cytometry of CD31^+^ cells in tumors from GSC1‐bearing mice. (J) Flow cytometry of CD31^+^ cells in tumors from GSC21‐bearing mice. (Date were presented as means ± standard deviations, * indicates *p* ≤ 0.05, ** indicates *p* < 0.01, **** indicates *p* < 0.0001.).

To verify how tumor cell expression of HEXB could affect ECs, GSC1 and GSC21 cells were treated with a HEXB lentivirus to overexpress HEXB (Figure S7A). Then conditional medium (CM) was collected to treat HBMECs in vitro. Immunofluorescence analysis revealed that the HBMECs treated with CM from HEXB‐overexpressing GSCs displayed decreased expression patterns of endothelial markers, and increased expression levels of mesenchymal markers (Figure S7B–D). Functionally, HBMECs pretreated with CM from HEXB‐overexpressing GSCs showed enhanced ability to migrate with increased monolayer permeability (Figure S7E–G). RNA sequencing was then performed to examine changes in ECs following treatment with HEXB‐overexpressing GSCs CM. The activity of 14 canonical signaling pathways were analyzed using the ssGSEA algorithm, indicating that Wnt signaling was activated in the ECs after HEXB‐overexpressing GSC1 cells CM treatment (Figure S7H and Table S3). The scRNA sequencing data also showed that the Wnt signaling pathway was enhanced in ECs following anti‐VEGF therapy (Figure 1F). These findings suggested that tumor‐expressing HEXB could activate the Wnt signaling in ECs, which thereby promoted Endo‐MT changes resulting in resistance to anti‐VEGF therapy. In contrast, treatment with CM from HEXB‐knockdown gliomaspheres strikingly impaired the Endo‐MT status, functionally abnormality and Wnt signaling in ECs (Figure 3D–H and Figure S8A–D).

Subsequently, we tested the effects of tumor‐expressed HEXB on Endo‐MT in vivo. We intracranially transplanted HEXB‐knockdown gliomaspheres into BALB/c nude mice. Flow cytometry was used to separate CD31^+^ ECs, after which Endo‐MT marker expression patterns were detected. The ratio of mesenchymal markers to endothelial markers was significantly decreased in the tumor‐derived CD31^+^ ECs in the HEXB‐knockdown group (Figure 3I,J and Figure S8E–G), revealing a reduced Endo‐MT status of the CD31^+^ ECs after HEXB suppression. Taken together, these data suggested that HEXB played a strong role in Endo‐MT in GBM.

### 3.5 IGFBP1 was Critical for HEXB‐Mediated Endo‐MT Changes

Because HEXB is a molecule with secretary function [12], we examined if tumor‐derived HEXB could directly regulate Endo‐MT changes in vitro. Gal‐PUGNAc, an inhibitor of HEXB that suppresses its enzymatic activity [14], was added into the CM of HEXB‐overexpressing gliomaspheres. Inhibiting HEXB in the CM had no effects on the HBMEC Endo‐MT status (Figure S9A). We next added recombinant human HEXB protein (rhHEXB) to the CM of HEXB‐knockdown gliomaspheres, which also resulted in no changes to the HBMEC Endo‐MT function (Figure S9B). Moreover, direct treatment of HBMECs with rhHEXB did not affect their Endo‐MT status (Figure S9C). These results suggested that tumor‐expressing HEXB participated in Endo‐MT in an indirect manner.

Next, we examined the roles and underlying mechanism of HEXB in the Endo‐MT process. To identify the angiogenic factors induced by HEXB in glioma cells, an array kit was used to detect the differentially expressed angiogenic factors in CM from the HEXB‐knockdown cells and control cells. Among these, IGFBP1 was the most downregulated angiogenic factor in the HEXB‐knockdown cells (Figure 4A and Figure S10A). Data from the CGGA, TCGA and GSE16011 databases demonstrated that GBM patients with high HEXB expression levels also had higher levels of IGFBP1 expression compared with patients with low HEXB expression patterns (Figure S10B). Furthermore, IGFBP1 expression and secretion levels were both significantly decreased by HEXB knockdown and upregulated by HEXB overexpression in both GSC1 and GSC21 cells (Figure 4B,C and Figure S10C).

**FIGURE 4:**
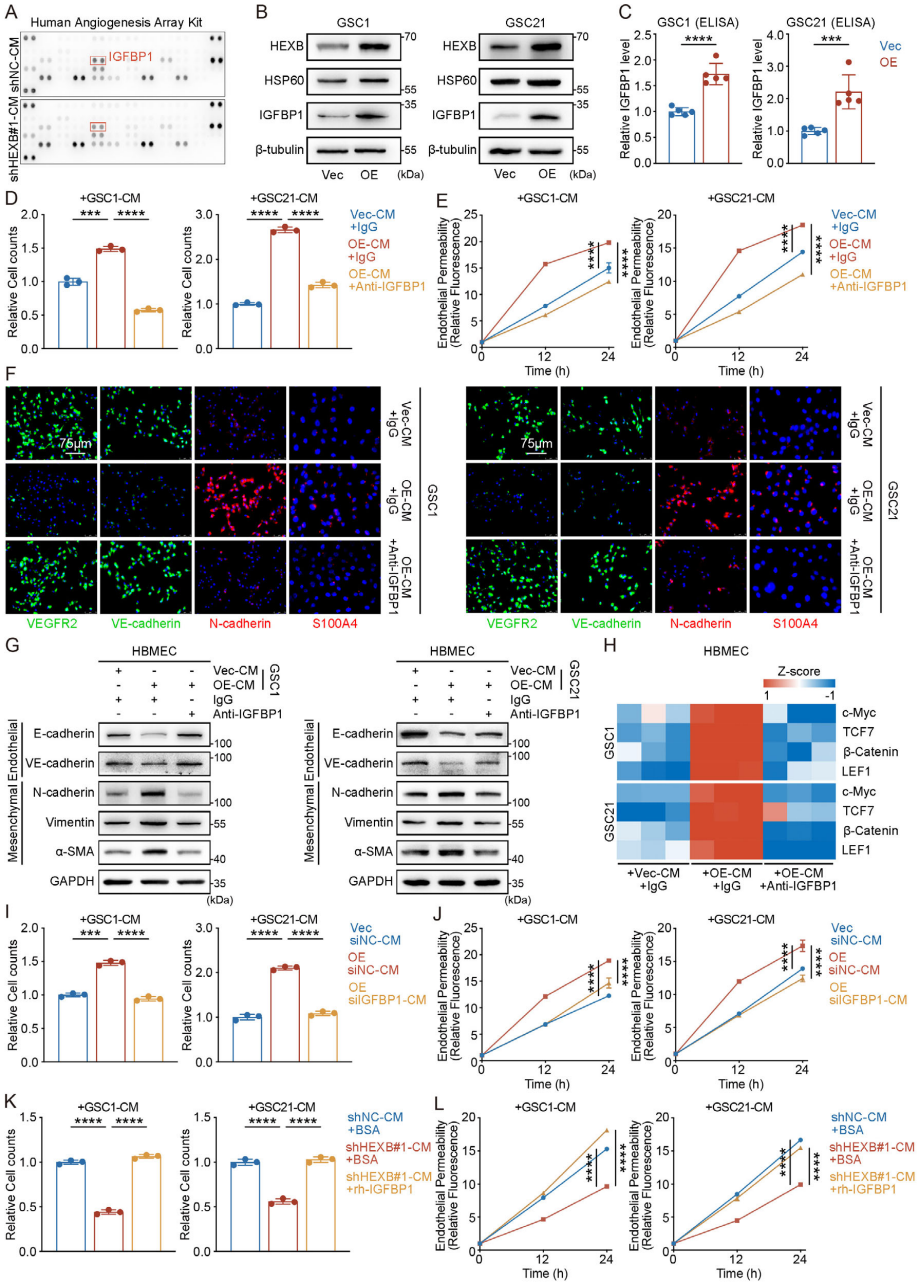
IGFBP1 played crucial role in HEXB‐mediated Endo‐MT changes of ECs. (A) Human angiogenesis Array result of conditional medium from GSC21 cells with HEXB knockdown or negative control. (B) Western blot results of GSCs after HEXB overexpression. (C) ELISA results of GSCs after HEXB overexpression. (D) The analysis of migration ability of HBMECs under different treatments (Anti‐IGFBP‐1 neutralizing antibody was added into CM at a dose of 10 µg/ml). (E) Permeability changes of HBMECs under different treatments (Anti‐IGFBP‐1 neutralizing antibody was added into CM at a dose of 10 µg/ml). (F) Immunofluorescence staining image of HBMECs under different treatments (Anti‐IGFBP‐1 neutralizing antibody was added into CM at a dose of 10 µg/ml). (G) Western blot results of HBMECs under different treatments (Anti‐IGFBP‐1 neutralizing antibody was added into CM at a dose of 10 µg/ml). (H) PCR results of HBMECs under different treatments (Anti‐IGFBP‐1 neutralizing antibody was added into CM at a dose of 10 µg/ml). (I) The analysis of migration ability of HBMECs treated with different CM. (J) Permeability changes of HBMECs treated with different CM. (K) The analysis of migration ability of HBMECs under different treatments (Recombinant IGFBP‐1 protein was added into CM at a dose of 1 µg/ml). (L) Permeability changes of HBMECs under different treatments (Recombinant IGFBP‐1 protein was added into CM at a dose of 1 µg/ml). (Date were presented as means ± standard deviations, *** indicates *p* < 0.001, **** indicates *p* < 0.0001.).

To further examine if HEXB‐induced Endo‐MT was mediated by IGFBP1, we used neutralizing antibodies to block IGFBP1 in the CM of HEXB‐overexpressing gliomaspheres. Elevated HEXB levels in the glioma cells resulted in substantially more Endo‐MT status, functionally abnormality and Wnt signaling in HBMEC. However, IGFBP1 blockage reduced this HEXB‐induced increase of this process (Figure 4D–H and Figure S10D–F). Likewise, we knocked down IGFBP1 expression in HEXB‐overexpressing gliomaspheres, then collected the CM. The enhanced Endo‐MT status, functionally abnormality and Wnt signaling in ECs induced by HEXB overexpression was reversed by IGFBP1 knockdown (Figure 4I,J and Figure S11). Recombinant human IGFBP1 (rhIGFBP1) was further added. Treatment of HBMECs with rhIGFBP1 not only promoted their Endo‐MT status (Figure S12), but also reversed the attenuated changes of Endo‐MT status, functionally abnormality and Wnt signaling activity caused by HEXB knockdown (Figure 4K,L and Figure S13). Collectively, these data suggested that IGFBP1 played a critical role in HEXB‐induced Endo‐MT changes.

### 3.6 HSP60 Played an Intermediary Role in the HEXB‐IGFBP1 Regulatory Mechanism

After confirming that HEXB could regulate the Endo‐MT process by modulating IGFBP1, we next aimed to identify the underlying molecular mechanism between HEXB and IGFBP1. Recognizing the central role of protein phosphorylation in tumor progression, we selected a comprehensive human phospho‐kinase array to capture a holistic view of the intracellular signaling cascades and essential stress response proteins modulated by HEXB deficiency. HSP60 was the most reduced protein in HEXB‐knockdown cells (Figure 5A). The HSP60 expression changes in GSCs were further confirmed by western blot analysis (Figure 4B). Second, we investigated if the effects of HEXB on IGFBP1 expression and Endo‐MT occurred via HSP60. Knocking down HSP60 expression using specific siRNAs resulted in significantly decreased IGFBP1 levels, as well as drastically attenuated the ability of HEXB to upregulate IGFBP1 levels (Figure 5B,C and Figure S14A). Next, HSP60 was knocked down in HEXB‐overexpressing gliomaspheres and the CM was collected. HSP60 knockdown prevented the effects of HEXB on HBMEC Endo‐MT status, functionally abnormality and Wnt signaling activity (Figure 5D–G and Figure S14B). Moreover, the gliomaspheres were treated with the HSP60 inhibitor mizoribine, which reduced IGFBP1 expression levels in a dose‐dependent manner (Figure S14C). The HEXB‐induced IGFBP1 expression, Endo‐MT status, functionally abnormality and Wnt signaling activity were all abrogated when HSP60 was inhibited with mizoribine (Figure 5H–K and Figure S14D–F). These findings indicated that the IGFBP1 expression induced by HEXB to promote Endo‐MT was regulated by HSP60.

**FIGURE 5:**
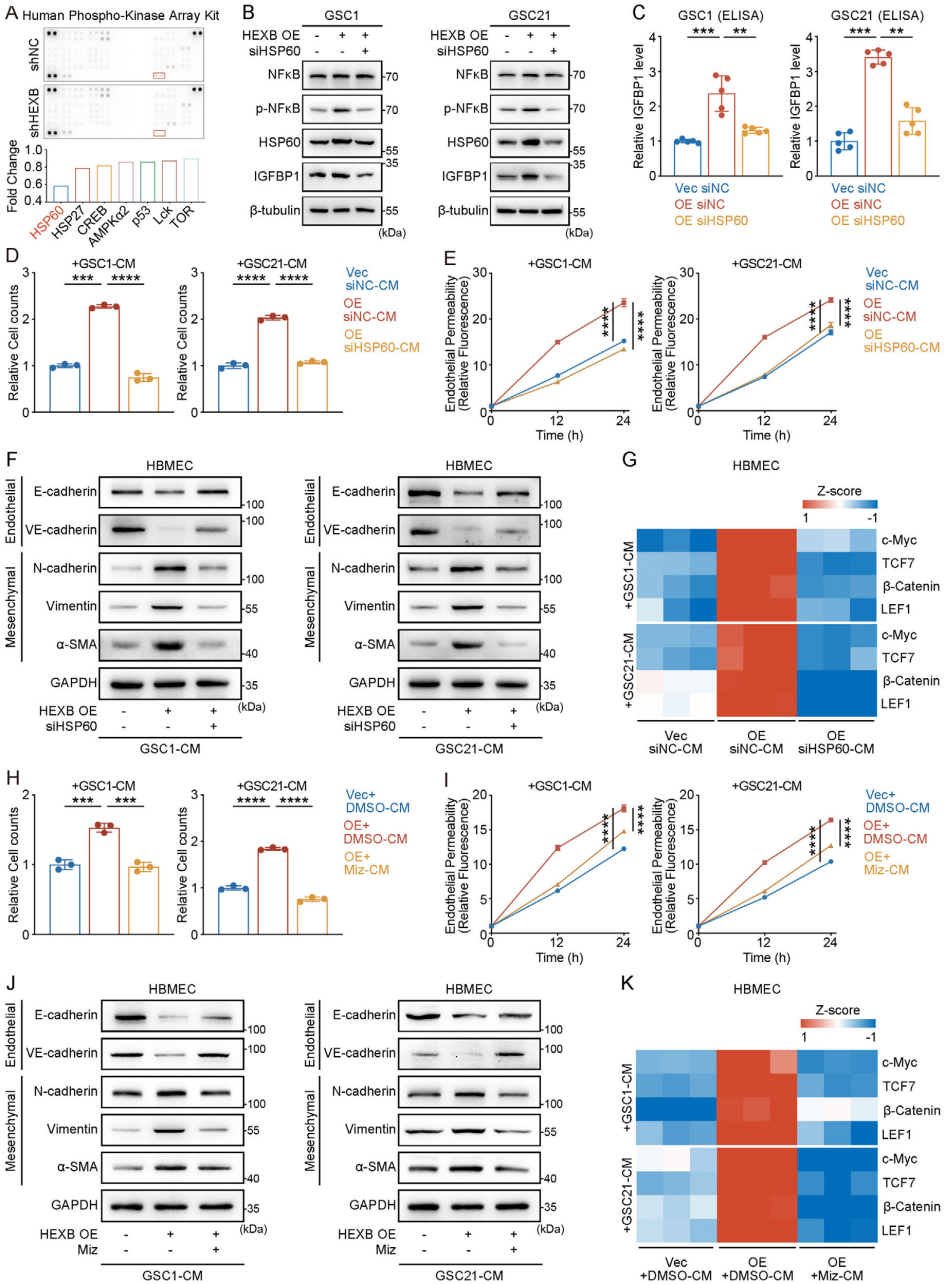
HEXB regulated IGFBP1 and Endo‐MT changes of ECs via HSP60. (A) Human Phospho‐Kinase Array result of GSC21 cells with HEXB knockdown or negative control. (B) Western blot results of GSCs under different treatments. (C) ELISA results of GSCs under different treatments. (D) The analysis of migration ability of HBMECs treated with different CM. (E) Permeability changes of HBMECs treated with different CM. (F) Western blot results of HBMECs treated with different CM. (G) PCR results of HBMECs treated with different CM. (H) The analysis of migration ability of HBMECs under different treatments (Miz was applied to indicated GSCs at a dose of 100 µM for 24 h prior to CM collection). (I) Permeability changes of HBMECs treated under different treatments (Miz was applied to indicated GSCs at a dose of 100 µM for 24 h prior to CM collection). (J) Western blot results of HBMECs under different treatments (Miz was applied to indicated GSCs at a dose of 100 µM for 24 h prior to CM collection). (K) PCR results of HBMECs under different treatments (Miz was applied to indicated GSCs at a dose of 100 µM for 24 h prior to CM collection). (Date were presented as means ± standard deviations, ** indicates*p* < 0.01, *** indicates*p* < 0.001, ^****^ indicates *p* < 0.0001.).

### 3.7 HEXB Directly Interacted with HSP60

To further explore the relationship between HSP60 and HEXB, we used proximity ligation assay (PLA) to examine their interaction in GSCs. Red signals represented the PLA fluorescent puncta indicating physical interaction, while blue signals represented DAPI nuclear. The results presented that HEXB could interacted with HSP60 (Figure 6A). Immunofluorescence assays also validated the co‐localization of HEXB and HSP60 staining, suggesting that these proteins potentially bind with each other (Figure S15A). Co‐IP assays were also performed in GSCs, showing that the anti‐HEXB antibody could pull down abundant HSP60 (Figure S15B). Similarly, the anti‐HSP60 antibody could pull down HEXB in these two cells (Figure S15B). We further performed co‐IP experiments using lysates of HEK293 cells transiently transfected with Flag‐tagged HEXB and Myc‐tagged HSP60 expression vectors. IP with an anti‐FLAG antibody pulled down Myc‐tagged HSP60, while IP with an anti‐Myc antibody pulled down Flag‐tagged HEXB, indicating that the two tagged proteins were associated with each other in HEK293 cells (Figure S15C). These data confirmed a direct interaction between HEXB and HSP60.

**FIGURE 6:**
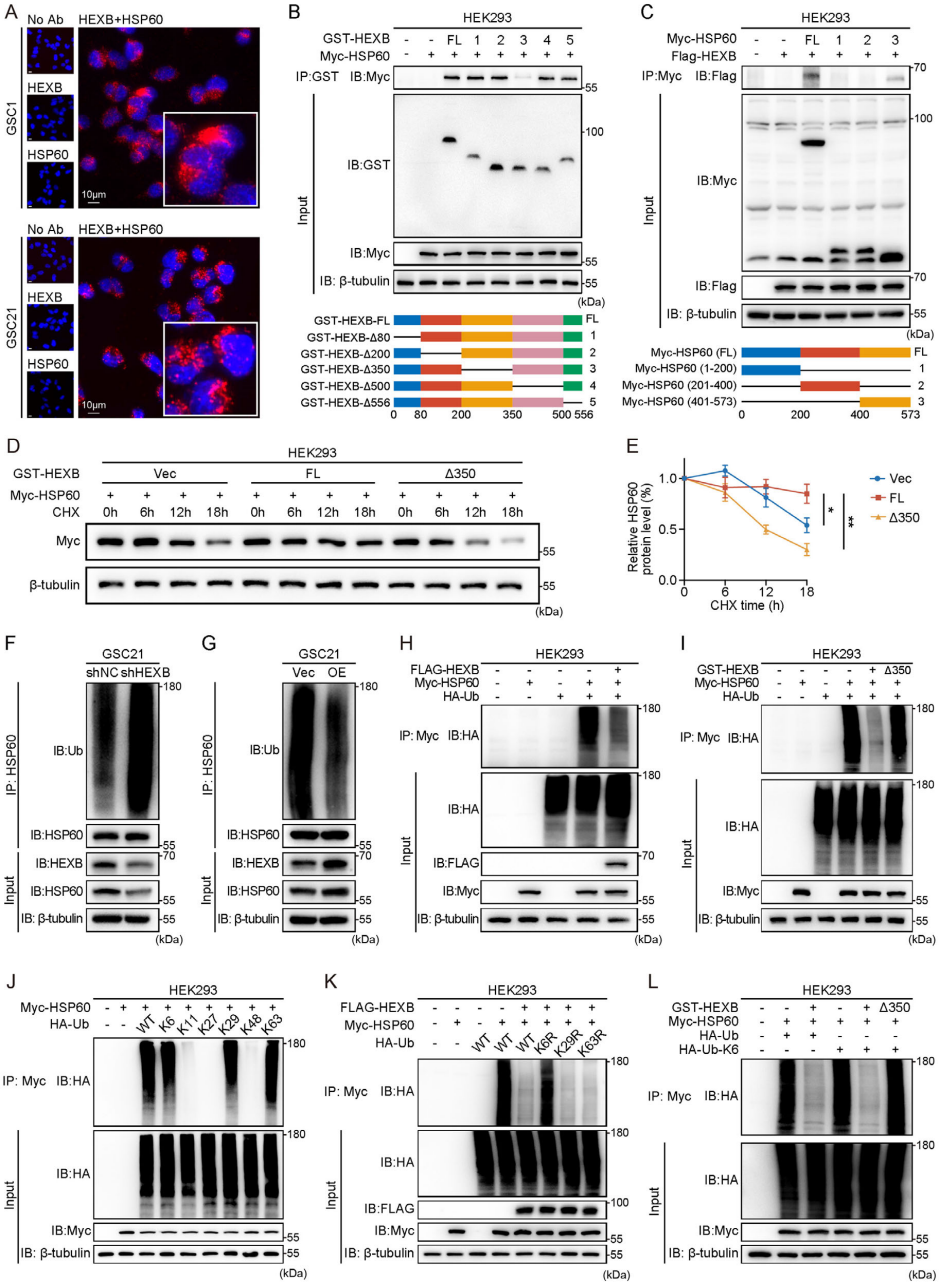
HEXB interacted with HSP60 and inhibited its ubiquitination process. (A) PLA analysis of HEXB and HSP60 in GSC1 and GSC21. Red signals represent the PLA fluorescent puncta indicating physical interaction. Blue signals represent DAPI nuclear staining. Either HEXB or HSP60 or no antibody (No Ab) was used as a negative control. (B) Western blot analysis of the direct interaction between different HEXB fragment and HSP60 by co‐immunoprecipitation in HEK293 cells. (C) Western blot analysis of the direct interaction between different HSP60 fragment and HEXB by co‐immunoprecipitation in HEK293 cells. (D and E) Representative western blot images and the degradation curve of time‐dependent Myc‐tagged HSP60 expression in HEK293 cells under different treatment. (F) Western blot analysis of immunoprecipitated HSP60 to determine the effect of HEXB knockdown on ubiquitination of HSP60 in GSC21. (G) Western blot analysis of immunoprecipitated HSP60 to determine the effect of HEXB overexpression on ubiquitination of HSP60 in GSC21. (H and I) Western blot analysis of the relationship between HEXB and HSP60 ubiquitination by co‐immunoprecipitation in HEK293 cells (CHX was applied to indicated cells at a dose of 100 µg/ml for different hours). (J) Western blot analysis of the interaction between HSP60 and different ubiquitin in HEK293 cells. (K) Western blot analysis of the effect of HEXB on the interaction between HSP60 and different ubiquitin in HEK293 cells. (L) Western blot analysis of the effect of different HEXB fragment on the interaction between HSP60 and different ubiquitin in HEK293 cells.

To investigate the functional domains responsible for their interaction, a series of deletion mutant constructs were designed for HEXB and HSP60. For HEXB, we constructed five truncated mutant plasmids and one wild‐type GST‐tagged plasmid. This series of GST‐tagged HEXB truncated plasmids and Myc‐tagged HSP60 plasmid were co‐transfected into HEK293 cells. Co‐IP experiments indicated that the HEXB‐Δ350 protein (deletion of amino acids 200–350) could no longer bind with HSP60 (Figure 6B). For HSP60, we constructed a vector that expressed the three peptides of the HSP60 protein from its crystal structure [15]. This series of Myc‐tagged HSP60 short peptides and GST‐tagged HEXB plasmids were co‐transfected into HEK293 cells. Co‐IP analysis showed that the HSP60 protein 401–573 amino acid fragment was necessary to pull down HEXB (Figure 6C). These results indicated the specific protein regions necessary for HEXB and HSP60 to directly interact.

We also detected the role of Gal‐PUGNAc in the interaction between HEXB and HPS60. Our molecular docking analysis (using MOE [16]) shows that Gal‐PUGNAc formed a stable interaction with Arg211 (Figure S15D). This site (Arg211) resided exactly within the amino acid 200–350 fragment of HEXB, the critical interaction region for binding HSP60 (Figure 6B and Figure S15E). Exactly, Co‐IP assays demonstrated that Gal‐PUGNAc treatment significantly reduced the binding between HEXB and HSP60 (Figure S15F and G). This indicated that Gal‐PUGNAc effectively disrupt the protein–protein interaction between HEXB and HSP60.

### 3.8 HEXB Inhibited HSP60 Degradation by Blocking Ubiquitination

After finding that HEXB and HSP60 could directly interact, we further explored if the protein stability of HSP60 was regulated by this interaction. After cycloheximide was used to block endogenous HSP60 protein synthesis, the HSP60 degradation rate was accelerated in HEXB‐knockdown gliomaspheres and restrained in HEXB‐overexpressing gliomaspheres (Figure S16A and B). Similar experiments were performed in HEK293 cells, where we further tested the effects of HEXB‐Full length (FL) and HEXB‐losing amino acids 200–350 (HEXB‐Δ350) on HSP60 protein stability. The HSP60 degradation rate was reduced in HEXB‐FL cells compared with negative control groups, while the changes in the HEXB‐Δ350 group were not significant (Figure 6D and E). These results suggested that the stability of HSP60 may be maintained by its direct interaction with HEXB.

We next used the proteasome inhibitor MG132 and the autophagy inhibitors 3‐MA and chloroquine to further explore the HSP60 degradation mechanism. MG132 treatment had no effects on HSP60 degradation, whereas the autophagy inhibitors 3‐MA and chloroquine significantly attenuated HSP60 degradation (Figure S16C and D). Therefore, we proposed that HEXB could maintain HSP60 stability by protecting it from degradation in a lysosome‐dependent manner.

Ubiquitination, one of the most common and important processes related to protein degradation, has been implicated in many tumor types, including glioma [17, 18]. We therefore examined if the ubiquitination of HSP60 was affected by HEXB. IP analysis indicated that loss of HEXB significantly increased the ubiquitination of endogenous HSP60 (Figure 6F), while HEXB overexpression reduced HSP60 ubiquitination levels (Figure 6G). Furthermore, HEXB overexpression reduced the ubiquitination of HSP60 in HEK293 cells, while the HEXB‐Δ350 mutant had no effect (Figure 6H and I). These findings confirmed that HEXB could block the ubiquitination of HSP60.

Ubiquitin molecules have several ubiquitin‐linked sites (K6, K11, K27, K29, K48, and K63) [19, 20]. To clarify the connection between HSP60 and ubiquitin, we transfected Myc‐HSP60 and different types of HA‐Ub molecules into HEK293 cells. The results showed that K6, K29, and K63 were the relevant ubiquitin binding sites for HSP60 (Figure 6J). We further constructed mutated HA‐Ub molecules (Ub‐K6R, Ub‐K29R, and Ub‐K63R) to analyze how HSP60 degradation was affected by HEXB. IP experiments revealed that only Ub‐K6R was associated with the significantly reduced ubiquitination of HSP60 resulting from its interaction with HEXB (Figure 6K). We next corroborated the role of Ub‐K6 in this mechanism. HSP60 degradation was existed in HEK293 cells transfected with Myc‐HSP60 and Ub‐K6, while 3‐MA and chloroquine attenuated HSP60 degradation in these cells (Figure S16E and F). IP experiments confirmed that Ub‐K6 could interact with HSP60, while this interaction was diminished with HEXB overexpression. However, HEXB‐Δ350 prevented HEXB from exerting these effects (Figure 6L). Overall, these findings indicated that HEXB could regulate HSP60 expression by preventing its degradation via blocking ubiquitination.

To elucidate the molecular identity of the specific E3 ubiquitin ligase responsible for the K6‐linked ubiquitination of HSP60, Co‐IP targeting HSP60 was performed in GSC1 cells, followed by liquid chromatography‐tandem mass spectrometry (LC‐MS/MS) to comprehensively map the HSP60 interactome. Concurrently, we established a list of E3 ligases documented to catalyze K6‐linked ubiquitination (Table S4). Intersecting our IP‐MS candidate dataset with this specialized E3 ligase list pinpointed TRIM21 as the most prominent candidate interacting partner (Figure S17A). To rigorously substantiate this finding, we performed a series of biochemical and functional validations. Co‐IP assays confirmed an interaction between HSP60 and TRIM21 in GSCs (Figure S17B). Overexpression of TRIM21 promoted the accumulation of K6‐linked ubiquitin chains on HSP60 (Figure S17C). These results established TRIM21 as the E3 ligase mediating the K6‐linked ubiquitination of HSP60. We next investigated how HEXB integrated into this process. Intriguingly, HEXB overexpression attenuated the association between HSP60 and TRIM21 (Figure S17D). Conversely, depletion of HEXB enhanced the HSP60–TRIM21 interaction, which consequently increased the K6‐linked ubiquitination of HSP60 and accelerated its subsequent degradation (Figure S17E). Collectively, these findings demonstrated that HEXB effectively shield HSP60 from lysosomal degradation by preventing aberrant TRIM21‐mediated K6‐linked ubiquitination, thereby maintaining its protein stability in GSCs.

### 3.9 The HEXB‐HSP60 Complex Regulated IGFBP1 Transcription via Activating NFκB Signaling

Because recent studies have illuminated that HSP60 knockdown can affect NFκB signaling activity [21], we first examined the effect of HSP60 on this pathway in our gliomaspheres. The NFκB phosphorylation levels were remarkably reduced with HSP60 deficiency, both with siRNA‐mediated knockdown and HSP60 inhibitor treatment (Figure S14A and C). HSP60 inhibition also attenuated the ability of HEXB to upregulate NFκB phosphorylation (Figure 5B and Figure S14D). Because NFκB can regulate IGFBP1 at the transcriptional level [22], we further investigated the role of NFκB signaling in regulating IGFBP1 expression. Gliomaspheres treated with the NFκB inhibitor BAY11‐7082 displayed downregulated IGFBP1 expression patterns in a dose‐dependent manner (Figure S18A and B). Furthermore, we examined the effect of NFκB signaling as the mediator between HEXB‐HSP60 and IGFBP1. Inhibiting NFκB signaling could reverse the promoting role of both HEXB overexpression and HSP60 overexpression on IGFBP1 mRNA and protein expression patterns (Figure S18C–F). Therefore, our data suggested that IGFBP1 can be regulated by the HEXB‐HSP60 complex through NFκB signaling.

### 3.10 IGFBP1 Activated Wnt Signaling in ECs through ITGA5/AKT/GSK3β

To delineate the precise regulatory link between IGFBP1 and Wnt signaling in HBMECs, we conducted a series of targeted mechanistic investigations. Because IGFBP1 possesses an RGD (Arg‐Gly‐Asp) motif, it functions as an integrin (such as Integrin α5) ligand [23], we first treated HBMECs with His‐tagged recombinant IGFBP1 protein and subsequently performed Co‐IP assays utilizing an anti‐His antibody to identify the specific cell‐surface receptor mediating IGFBP1 function. The results demonstrated that IGFBP1 physically and specifically binds to the integrin receptor ITGA5 in HBMECs (Figure S19A). For Wnt signaling, active GSK3β maintains the β‐catenin “destruction complex”, marking β‐catenin for degradation. However, the phosphorylation of GSK3β (at Ser9) by AKT catalytically inactivates it [24]. Since IGFBP1/ITGA5 can activate phosphorylation of AKT (Figure S19B), the IGFBP1‐induced AKT signal structurally disrupts the destruction complex, allowing β‐catenin to stabilize and drive the expression of downstream Wnt targets (e.g., TCF7/LEF1, c‐Myc). To prove that ITGA5 is the obligatory upstream mediator responsible for initiating this specific signal, we demonstrated that knocking down ITGA5 blunts both the AKT/GSK3β phosphorylation and the subsequent Wnt pathway activation caused by IGFBP1 (Figure S19C and D). Finally, to firmly establish AKT as the essential molecular bridge in this process, we found that pharmacological inhibition of AKT by MK‐2206 effectively abolishes the IGFBP1‐induced GSK3β phosphorylation and prevents the subsequent Wnt activation (Figure S19E and F). Collectively, these sequential experimental findings validate a definitive IGFBP1‐ITGA5‐AKT‐GSK3β‐Wnt signaling axis, providing a comprehensive molecular basis for IGFBP1‐driven Endo‐MT process.

### 3.11 HEXB Inhibition Demonstrated Satisfactory Therapeutic Effects and Reversed Anti‐VEGF Therapy Resistance in GBM

Considering the promoting roles of HEXB in the Endo‐MT process and GBM malignant progression, we investigated the therapeutic effects of pharmacological inhibition of HEXB in GBM in vivo. We first demonstrated that administration of Gal‐PUGNAc, a selective inhibitor of HEXB, significantly prolonged the survival of SB‐bearing mice (Figure 7A). Histopathological analysis via H&E staining further confirmed that Gal‐PUGNAc treatment effectively restricted tumor burden (Figure 7B). To elucidate the cellular mechanisms underlying this therapeutic efficacy, we characterized the Endo‐MT status within the GBM tissues using multi‐color immunofluorescence staining. Compared with control group, Gal‐PUGNAc treatment significantly contracted the population of mesenchymal‐like CD31^+^S100A4^+^ ECs, while restoring functional CD31^+^VEGFR2^+^ EC population (Figure 7C and Figure S20A). Given that the reversal of Endo‐MT is typically associated with the recovery of endothelial functional integrity, we then evaluated the perfusion and structural properties of the intratumoral vessels as functional readouts. We assessed vessel perfusion by co‐staining lectin with CD31 to calculate the vessel perfusion rate (defined as the percentage of lectin^+^ area over CD31^+^ area). Notably, the vessel perfusion rate was significantly increased under Gal‐PUGNAc treatment (Figure 7D). Morphological assessment of the vessel lumens revealed that vessels in control group exhibited typical pathological disintegration such as aberrant dilation or focal occlusion, while the functional vessels in Gal‐PUGNAc group displayed highly organized, continuous vascular architectures with clearly defined, patent lumens (Figure 7D). These findings were fully recapitulated in a U87 xenograft mouse model, demonstrating that in vivo HEXB inhibition successfully reverses the Endo‐MT process, thereby restoring the physiological and functions of ECs in GBM tissues (Figure 7E–H and Figure S20B).

**FIGURE 7:**
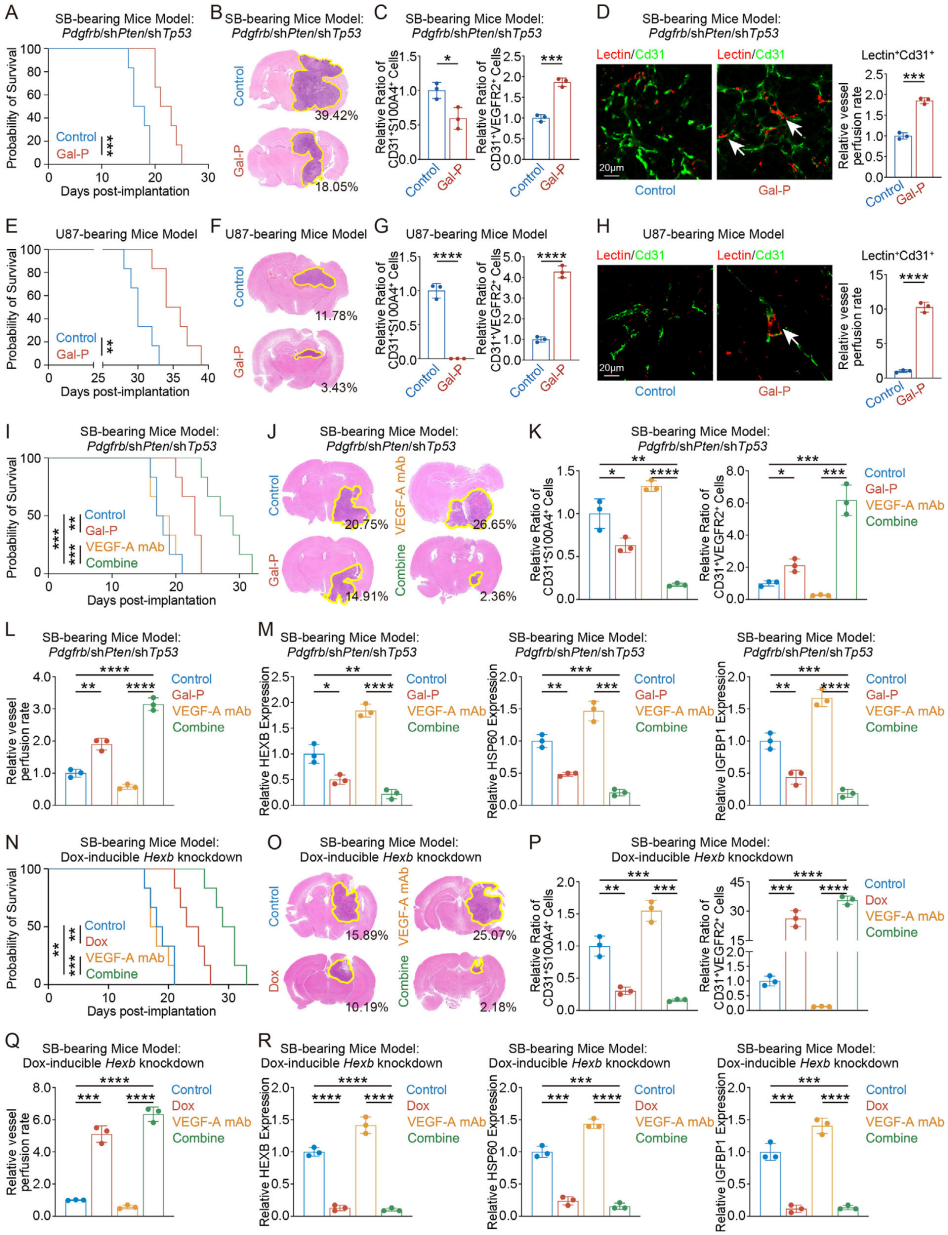
HEXB inhibition improved anti‐VEGF treatment efficacy. (A) Survival of SB‐bearing C57BL6 mice under different treatments (log‐rank test, n = 6). (B) The images of H&E staining of tumors from SB‐bearing C57BL6 mice under different treatments (numbers means the ratio of tumor area in whole brain). (C) The analysis of three‐color immunofluorescence staining of tumors in different treatment group. (D) The analysis and representative images of lectin‐positive vessels in total vessels under different treatment. (E) Survival of U87‐bearing BALB/c nude mice under different treatments (log‐rank test, n = 6). (F) The images of H&E staining of tumors from U87‐bearing BALB/c nude mice under different treatments (numbers means the ratio of tumor area in whole brain). (G) The analysis of three‐color immunofluorescence staining of tumors in different treatment group. (H) The analysis and representative images of lectin‐positive vessels in total vessels under different treatment. (I) Survival of SB‐bearing C57BL6 mice under different treatments (log‐rank test, n = 6). (J) The images of H&E staining of tumors from SB‐bearing C57BL6 mice under different treatments (numbers means the ratio of tumor area in whole brain). (K) The analysis of three‐color immunofluorescence staining of tumors in different treatment group. (L) The analysis of lectin‐positive vessels in total vessels under different treatment. (M) The analysis of HEXB, HSP60 and IGFBP1 expression in tumors under different treatment. (N) Survival of SB‐bearing C57BL6 mice under different treatments (log‐rank test, n = 6). (O) The images of H&E staining of tumors from SB‐bearing C57BL6 mice under different treatments (numbers means the ratio of tumor area in whole brain). (P) The analysis of three‐color immunofluorescence staining of tumors in different treatment group. (Q) The analysis of lectin‐positive vessels in total vessels under different treatment. (R) The analysis of HEXB, HSP60 and IGFBP1 expression in tumors under different treatment. (Date were presented as means ± standard deviations, * indicates *p* ≤ 0.05, ** indicates *p* < 0.01, *** indicates *p* < 0.001, **** indicates *p* < 0.0001.).

Because of the potential role of the Endo‐MT process in anti‐VEGF therapy resistance, we next investigated whether HEXB inhibition could overcome this resistance and improve the efficacy of anti‐VEGF treatment in vivo. The murine spontaneous GBM model generated with the SB system was induced in C57BL/6 mice, followed by single or combined treatment with Gal‐PUGNAc and VEGF‐A antibody. Consistent with the established resistance to anti‐angiogenic therapies, VEGF‐A antibody did not significantly prolong mouse survival or limit tumor growth. however, the combination therapy approach markedly enhanced mice survival and robustly restricted tumor growth (Figure 7I and J). To determine if these synergistic therapeutic benefits were indeed mediated by the modulation of endothelial Endo‐MT and subsequent functional states, we examined intratumoral vessel marker expressions and functions. Under VEGF‐A antibody treatment, ECs exhibited a pronounced Endo‐MT state, as manifested by an elevated proportion of mesenchymal‐like CD31^+^S100A4^+^ ECs and a reduced proportion of functional CD31^+^VEGFR2^+^ ECs. In contrast, the combination treatment successfully reversed this treatment‐induced Endo‐MT, leading to a low Endo‐MT state (Figure 7K and Figure S21A). Corresponding to this molecular reversal, we observed a substantial improvement in vascular function, characterized by an increased vessel perfusion rate and well‐organized, patent vascular structures in combine group (Figure 7L and Figure S21B). Downstream of this cascade, IHC staining showed that Gal‐PUGNAc decreased the expression of HEXB, HSP60 and IGFBP1 in tumors, with the combination treatment yielding the lowest expression levels (Figure 7M and Figure S21C). We also investigated the biosafety of Gal‐PUGNAc combined with anti‐VEGF therapy. H&E staining of heart, liver, spleen, lung and kidney was performed, revealing that administering Gal‐PUGNAc alone or in combination with VEGF‐A antibody in vivo did not affect the tissue structure or cause damage to mouse body (Figure S21D). Finally, to confirm that these therapeutic and cellular effects were specifically attributable to HEXB depletion, we established a doxycycline (Dox)‐inducible Hexb knockdown SB model. Upon Dox administration, genetic ablation of Hexb fully phenocopied the effects of Gal‐PUGNAc, prolonging survival, suppressing tumor growth, inhibiting Endo‐MT, and restoring EC function, thereby sensitizing GBM tumors to anti‐VEGF therapy (Figure 7N–R and Figure S22). Collectively, these results indicate that HEXB inhibition serves as a powerful strategy to sensitize GBM to anti‐VEGF treatment by reversing Endo‐MT and restoring endothelial function, culminating in highly synergistic therapeutic efficacy in preclinical models.

## 4 Discussion

Prominent vascularity is a hallmark of tumor development, progression, and treatment resistance [25]. However, conventional anti‐angiogenic treatment approaches targeting the VEGF pathway have presented many difficulties and failures in certain tumor types, including GBM [4]. In the present study, we established a mechanistic and translational framework for anti‐VEGF resistance in GBM centered on endothelial plasticity and tumor–endothelial cross talk. We identified HEXB as a tumor intrinsic, anti‐VEGF inducible driver that promoted Endo‐MT process via a HEXB/HSP60/NFκB/IGFBP1 signaling cascade. Pharmacologic HEXB inhibition reversed Endo‐MT, and cooperated with anti‐VEGF blockade to yield superior antitumor effects. These findings converge on a model in which anti‐VEGF pressure triggers a tumor cell program that reprograms ECs toward mesenchymal states, resulting in vascular adaptation and therapeutic escape.

Although anti‐VEGF therapy was initially considered promising for GBM, its clinical efficacy is severely hampered by adaptive resistance, primarily driven by EC plasticity and the Endo‐MT process [6, 26, 27]. In this study, we established a mechanistic and translational framework for this resistance, identifying HEXB as a tumor‐intrinsic, anti‐VEGF‐inducible driver of Endo‐MT. By upregulating IGFBP1 secretion, HEXB domesticates ECs to undergo mesenchymal transformation, bypassing canonical VEGF pathways and enabling therapeutic escape. Crucially, clinical data corroborates that GBM patients with high HEXB expression exhibit worse survival outcomes following bevacizumab treatment. These findings helped identify HEXB as a potential target for improving anti‐VEGF therapeutic efficacy in GBM.

Mechanistically, we delineate a novel HEXB/HSP60/NFκB/IGFBP1 signaling cascade. A key mechanistic insight of our study is that HEXB directly binds and stabilizes HSP60 by inhibiting its ubiquitin‐mediated degradation. Ubiquitination is an extremely important example of a post‐translational modification with a critical function in glioma progression [18, 28, 29]. Ubiquitination of proteins can affect their activities and drastically change their function [30, 31]. The ubiquitination of HSP60 has not been investigated extensively, while there is abundant information regarding the effects of ubiquitination on other HSP family members, such as HSP70 and HSP90 [32, 33, 34]. Because HEXB is a glycosyl hydrolase rather than a canonical E3 ubiquitin ligase or deubiquitinase, our findings suggest that HEXB competitively blocks ubiquitin ligase binding to HSP60. This aberrant stabilization of HSP60 is essential for the sustained activation of NFκB and subsequent IGFBP1 secretion, ultimately precipitating the Endo‐MT process in the tumor microenvironment.

Addressing the clinical need to overcome anti‐VEGF resistance, our data highlights HEXB as a tractable therapeutic vulnerability. Pharmacological inhibition of HEXB using Gal‐PUGNAc effectively reversed the Endo‐MT process and restrained tumor progression in spontaneous GBM mouse models. Most notably, combining Gal‐PUGNAc with anti‐VEGF blockade achieved profound synergistic anti‐tumor effects, restoring endothelial‐like states and extending survival. Crucially, within the broader context of the tumor microenvironment (TME), the benefits of targeting HEXB likely extend beyond direct effects on endothelial plasticity. As suggested by our prior work, dampening HEXB may additionally reprogram tumor‐associated macrophages (TAMs) away from an immunosuppressive and pro‐angiogenic M2 phenotype [12, 35]. This dual action, restoring endothelial normalization while concurrently remodeling immune infiltrates, positions HEXB as a critical node in orchestrating a therapy‐responsive TME.

Despite these promising findings, we acknowledge certain limitations in the present study. First, our translational insights currently rely on a restricted number of clinical cases, necessitating future validation in larger, independent GBM cohorts to firmly establish the prognostic value of HEXB. Second, while the pharmacological inhibition of HEXB using Gal‐PUGNAc yielded substantial synergistic effects in vivo, its application in our murine models remains preliminary. Moving toward clinical translation, the development of next‐generation HEXB inhibitors must prioritize target selectivity, safety profiles, and blood‐brain barrier (BBB) penetrance, alongside evaluating their pharmacokinetics with standard chemoradiation. In conclusion, our study uncovers a tumor‐intrinsic mechanism of vascular adaptation under anti‐angiogenic pressure. By delineating the HEXB/HSP60/IGFBP1 axis in driving Endo‐MT, we nominate dual HEXB and VEGF blockade as a rationally designed strategy to overcome treatment resistance in GBM.

## Author Contributions

**Tianqi Liu**, **Xin Chen**, **Tao Sun**, **Jianqi Wu**, **Wen Cheng**, and **Anhua Wu** contributed to the study conceptualization; **Tianqi Liu**, **Xin Chen**, **Jianqi Wu**, **Wen Cheng**, and **Anhua Wu** secured funding for the project; **Tianqi Liu**, **Xin Chen**, and **Tao Sun** conducted the experiments with assistance from **Haixu Yan**, **Xing Liu**, **Hongda Lin**, **Xiangyu Wang**, **Ziao Xiao**, **Yang Xu**, and **Qiang Fu**; **Jianqi Wu** and **Peng Cheng** provided technical support; **Tianqi Liu** and **Anhua Wu** wrote the manuscript. All authors approved final manuscript.

## Funding

This work was supported by the National Natural Science Foundation of China (No. Nos. 82573052 and 82303346 to T. Liu; No. 82472833 to A. Wu; No. 82373342 to W. Cheng; No. 82403452 to X. Chen; No. 82303837 to J. Wu); the Science and Technology Planning Project of Liaoning Province (No. 2021JH1/10400049 to A. Wu); the Joint Project of Liaoning Province (No. 2023JH2/101700168 to T. Liu and No. 2023JH2/101700202 to W. Cheng); the Shengjing Hospital of China Medical University 345 Talent Project (No. 1000801592 to W. Cheng).

## Conflicts of Interest

The authors declare no conflicts of interest.

## Supporting information




**Supporting File 1**: advs78208‐sup‐0001‐SuppMat.pdf.

**Supporting File 2**: advs78208‐sup‐0002‐SuppMat.xlsx.

## Data Availability

The data that support the findings of this study are available from the corresponding author upon reasonable request.
