# Supplementary material for: Tumor‐Intrinsic HEXB Drives Endothelial‐To‐Mesenchymal Transition and Confers Resistance to Anti‐Angiogenic Therapy in Glioblastoma
Source: Adv Sci (Weinh). 2026 Oct 8:e78208. Online ahead of print. doi: 10.1002/advs.78208 (PMC13650030; doi:10.1002/advs.78208)
Supplement: Supplementary file 1 [file ADVS-9999-e78208-s002.pdf]

Supporting Information

Tumor-Intrinsic HEXB Drives Endothelial-to-Mesenchymal Transition and Confers Resistance to Anti-Angiogenic Therapy in Glioblastoma

Tianqi Liu[1], Xin Chen[1], Tao Sun[1], Jianqi Wu[1], Haixu Yan[1], Xing Liu[1,2], Hongda Lin[1], Xiangyu Wang[1], Ziao Xiao[1], Yang Xu[1,3], Qiang Fu[1], Peng Cheng[2], Wen Cheng[1, *], Anhua Wu[1, *]

[1] Department of Neurosurgery, Shengjing Hospital of China Medical University, Shenyang, Liaoning, China

[2] Department of Neurosurgery, The First Hospital of China Medical University, Shenyang, Liaoning, China

[3] Department of Rehabilitation, Shengjing Hospital of China Medical University, Shenyang, Liaoning, China

Tianqi Liu, Xin Chen, Tao Sun, Jianqi Wu contributed equally to this work.

Correspondence: Wen Cheng (cmu071207@163.com), Anhua Wu (ahwu@cmu.edu.cn).

List of Supporting Information File

Figure S1 to S22 for multiple Supplementary Figures

Table S1 to S4 for multiple Supplementary Tables

Supplementary Figure 1

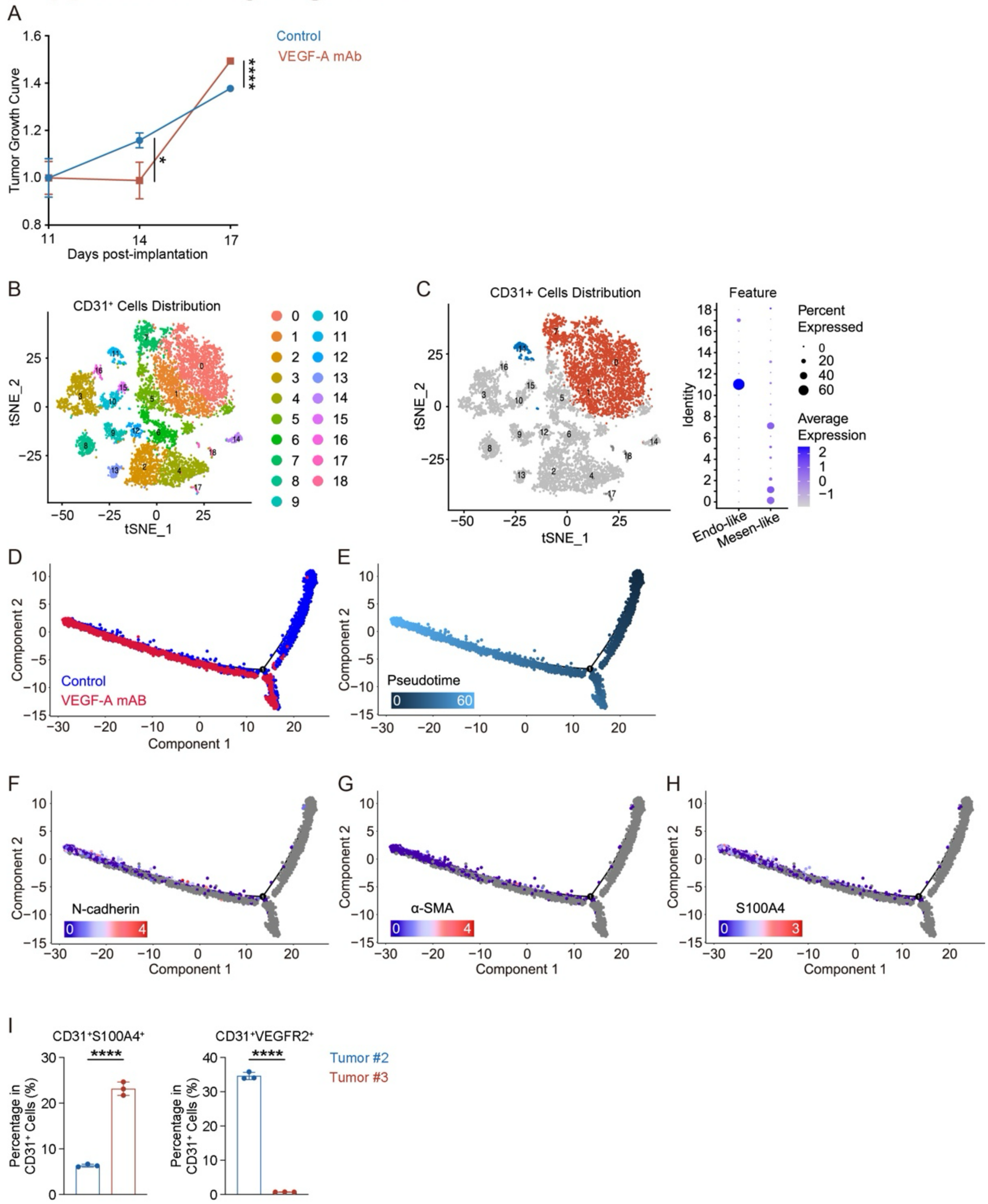

Figure S1. Endo-MT process presented the failure of anti-VEGF therapy.

(A) Quantitative tumor growth curve of SB-bearing C57BL6 mice treatment with IgG isotype control antibody (control group) or a VEGF-A antibody (VEGF-A mAb group); (B) t-Distributed Stochastic Neighbor Embedding (tSNE) visualization colored according to clusters of $CD31^+$ cells; (C) The single cells from SB-bearing mice tumors were clustered into endothelial-like cells, intermediated-like cells and mesenchymal-like cells; (D-H) Trajectoy analysis using Monocle2. $CD31^+$ Cells were highlighted according to treatment groups (D), pseudotime (E) or expression of Endo-MT marker genes (F-H); (I) The analysis of three-color immunofluorescence staining of clinical tumors in different treatment group.

(Date were presented as means ± standard deviations, ns indicated P > 0.05, *

indicates P ≤ 0.05, **** indicates P < 0.0001.)

Supplementary Figure 2

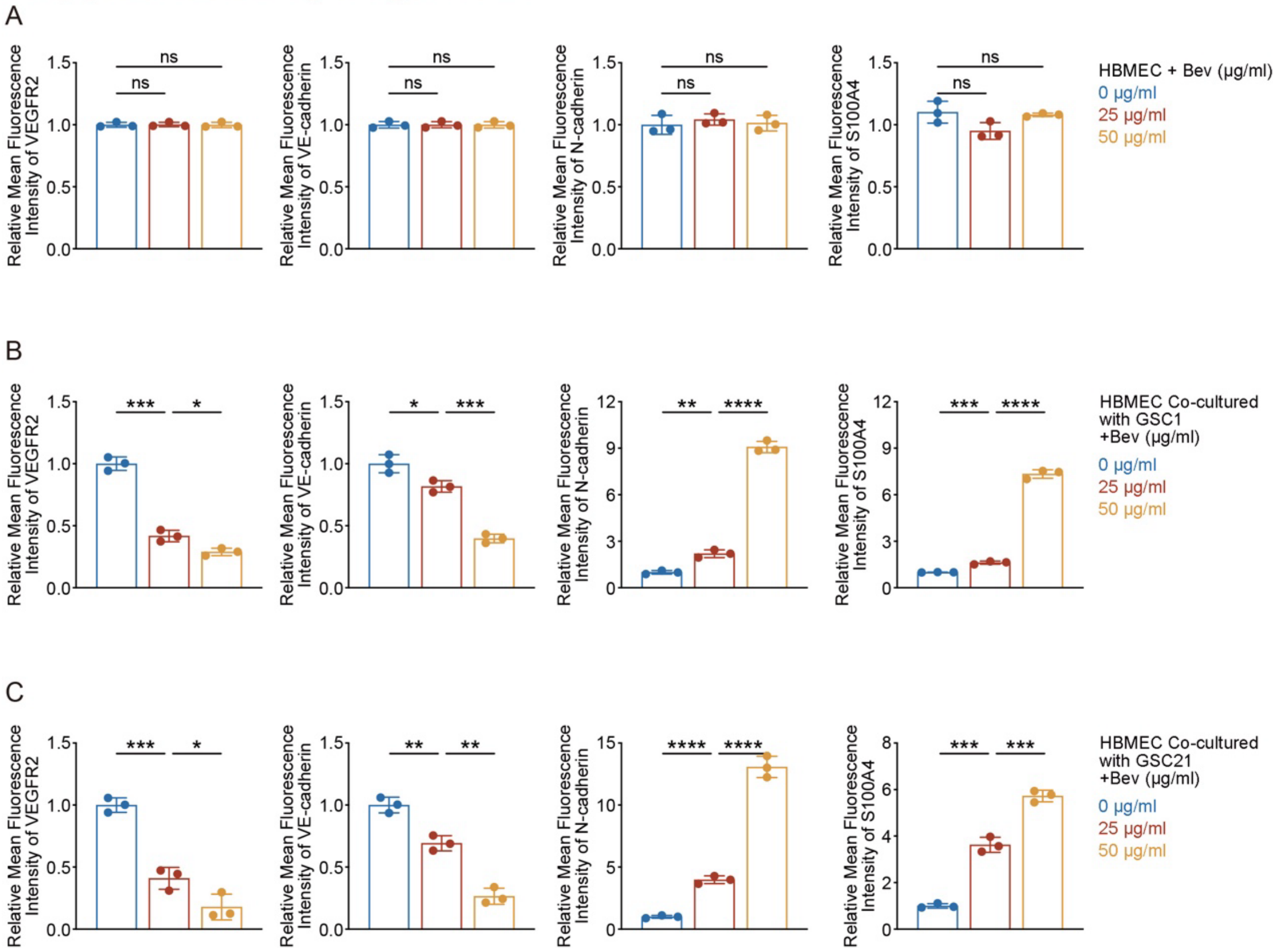

Figure S2. Statistical analysis of immunofluorescence staining of HBMEC under different treatments.

(A) Statistical analysis of immunofluorescence staining of HBMEC treated with different dose of bevacizumab; (B) Statistical analysis of immunofluorescence staining of HBMEC co-cultured with GSC1 treated with different dose of bevacizumab; (C) Statistical analysis of immunofluorescence staining of HBMEC co-cultured with GSC21 treated with different dose of bevacizumab.

(Date were presented as means ± standard deviations, ns indicated P > 0.05, * indicates P ≤ 0.05, ** indicates P < 0.01, *** indicates P < 0.001, **** indicates P < 0.0001.)

Supplementary Figure 3

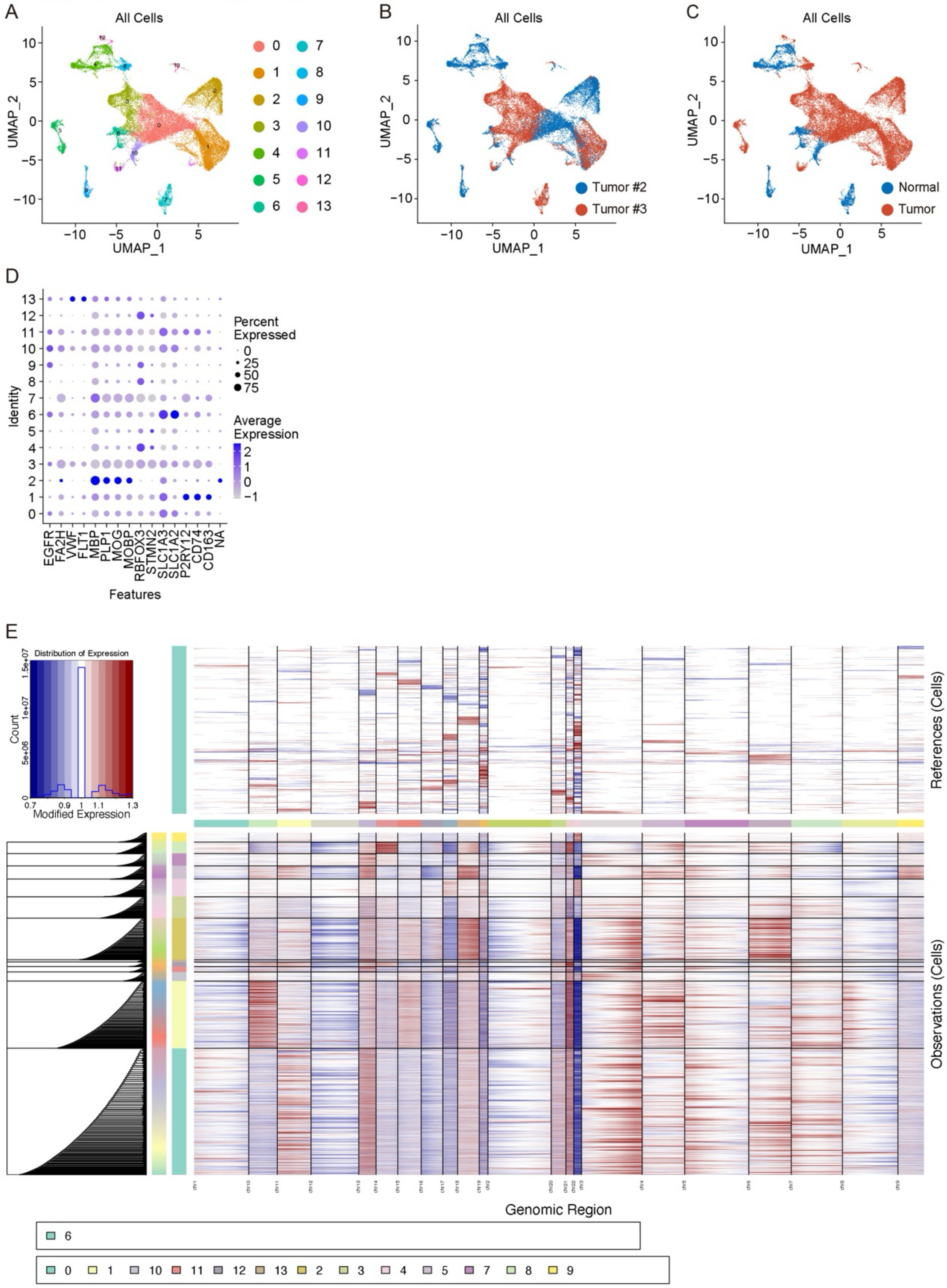

Figure S3. Single-nuclei transcriptomic analysis distinguishing tumor cells from normal cells in two human GBM samples.

(A) Uniform Manifold Approximation and Projection (UMAP) plot displaying the integrated cell map, which consists of 14 cell clusters. Cells are colored by clusters; (B-C) Patients' (B) or cell types' (C) annotation of all clustered cells; (D) Dot plot showing representative marker genes across cell clusters. Dot size is proportional to

the fraction of cells expressing specific genes. Color intensity corresponds to the relative expression of specific genes; (E) CNV inferred by snRNA-seq in two human GBM samples to screen tumor cells.

Supplementary Figure 4

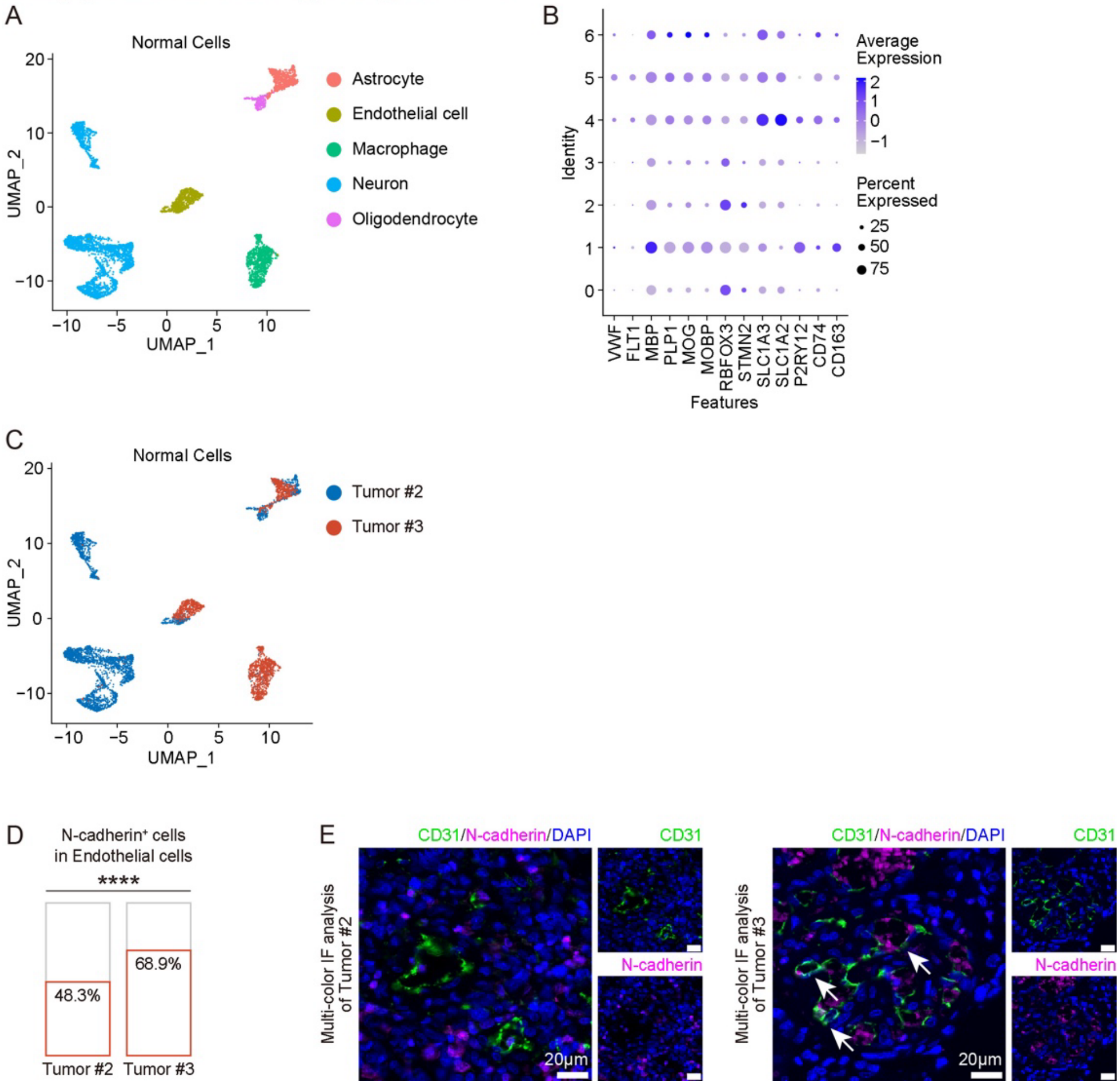

Figure S4. Single-nuclei transcriptomic analysis and clinical immunofluorescence staining of two human GBM samples.

(A) UMAP plot displaying the integrated cell map, which consists of 5 cell types. Cells are colored by cell types; (B) Dot plot illustrating the average expression and frequency of representative marker genes in the subclusters of normal cells; (C) Samples' annotation of all clustered normal cells; (D) The proportion of N-cadherin^{+} cells in endothelial cells; (E) Immunofluorescence staining image of different clinical GBM samples.

(Date were presented as means ± standard deviations, **** indicates $P < 0.0001$.)

Supplementary Figure 5

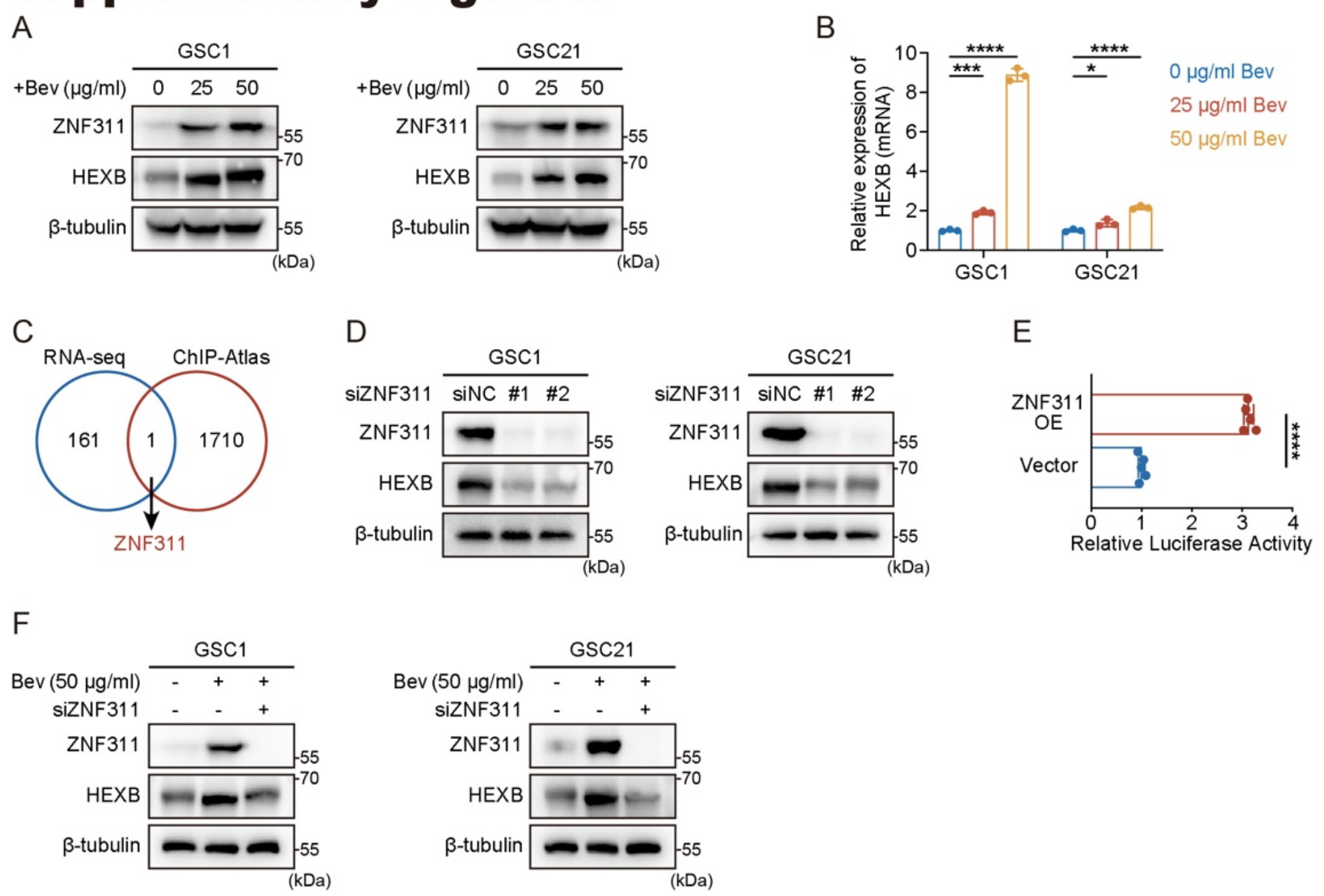

Figure S5. Bevacizumab upregulated HEXB expression via ZNF311 in GSCs.

(A) Western blot analysis of ZNF311 and HEXB protein levels in GSCs after different doses of bevacizumab treatment; (B) PCR results of HEXB expression in GSCs after different doses of bevacizumab treatment; (C) Venn diagram illustrating the intersection of candidate genes identified ZNF311 as the overlapping target; (D) Western blot analysis of ZNF311 and HEXB protein expression in GSCs under different treatments; (E) Relative luciferase reporter activity in HEK293 cells; (F) Western blot analysis of ZNF311 and HEXB protein levels in GSCs under different treatments.

(Date were presented as means ± standard deviations, * indicates $P < 0.05$, *** indicates $P < 0.001$, **** indicates $P < 0.0001$.)

Supplementary Figure 6

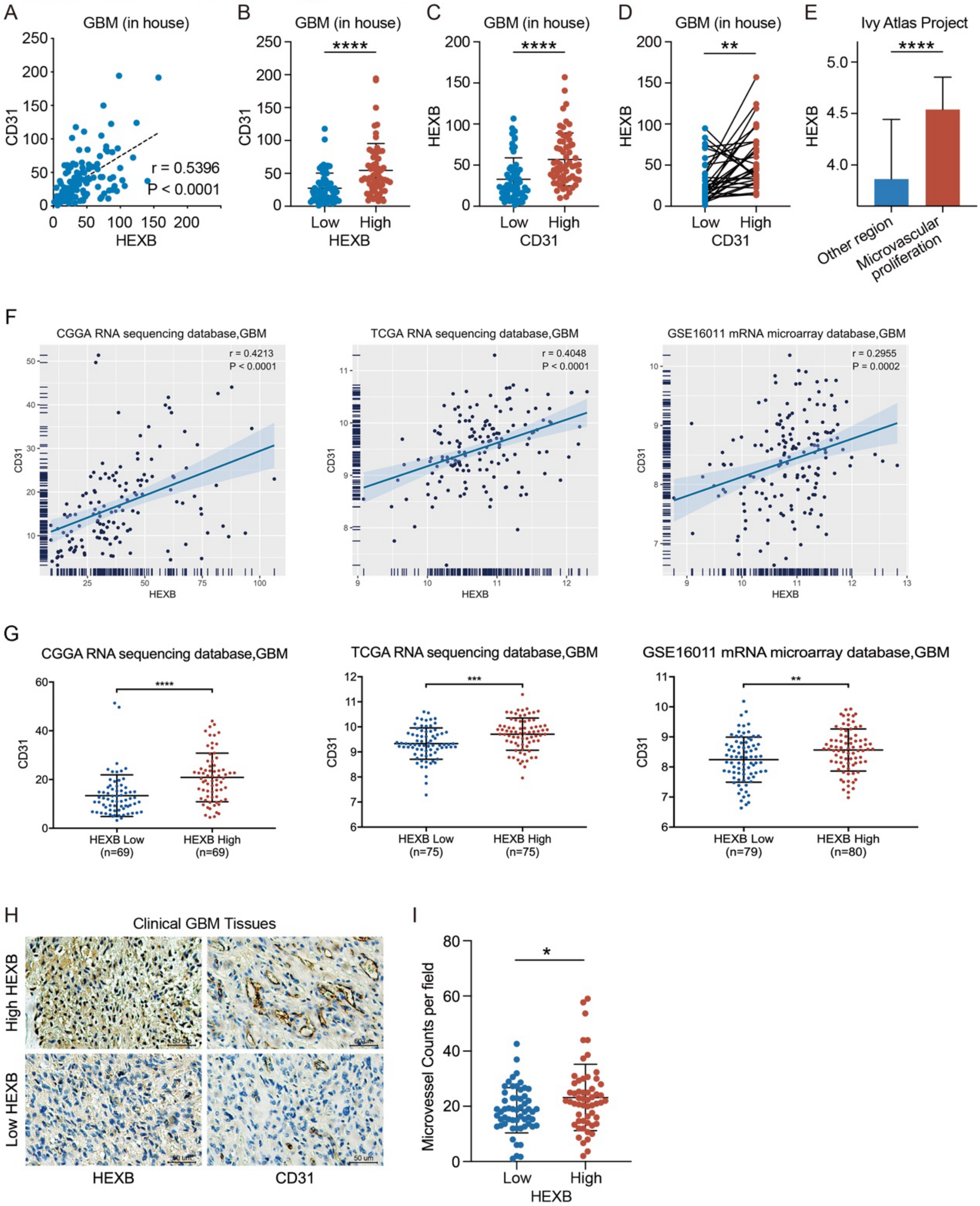

Figure S6. HEXB was strongly correlated with endothelial cells.

(A) The relationship between HEXB and CD31; (B) The expression of CD31 (endothelial cell marker) in GBM patients with high HEXB group and low HEXB group; (C) The expression of HEXB in GBM patients with high CD31 group and low CD31 group; (D) The differences of HEXB between the samples with highest CD31 and the samples with lowest CD31 in each patient; (E) HEXB expression in Ivy Atlas Project; (F) The relationship between HEXB and CD31 in CGGA, TCGA and GSE16011 GBM cohorts; (G) The expression of CD31 in GBM patients with high HEXB group and low HEXB group in CGGA, TCGA and GSE16011 GBM cohorts; (H and I) IHC staining of HEXB and CD31 expression in clinical GBM samples.

(Date were presented as means ± standard deviations, * indicates P ≤ 0.05, ** indicates P < 0.01, *** indicates P < 0.001, **** indicates P < 0.0001.)

Supplementary Figure 7

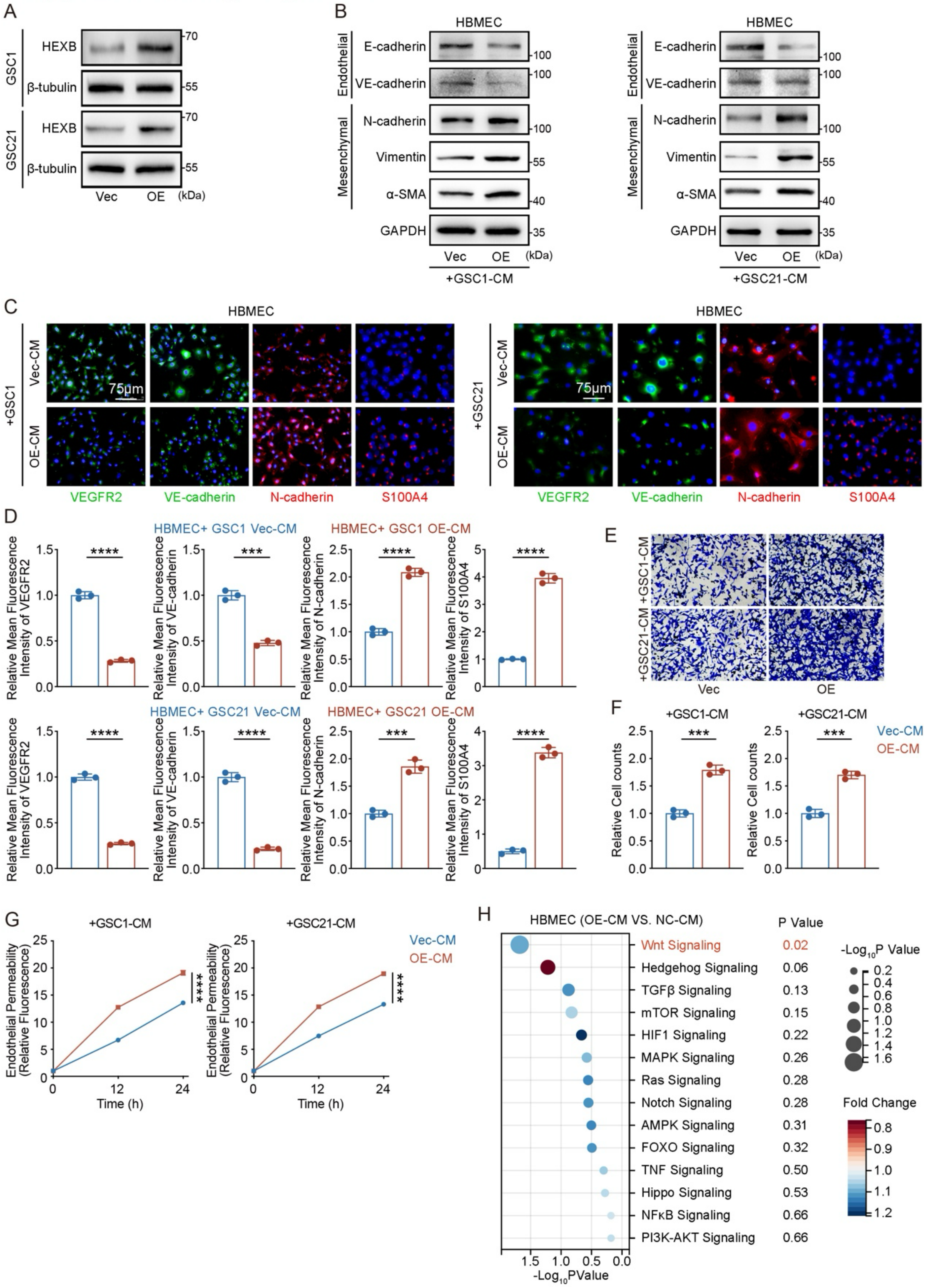

Figure S7. HEXB promoted the Endo-MT status of ECs.

(A) Western blot results of gliomaspheres transfected with HEXB overexpressed lentivirus; (B) Western blot results of HBMECs under different treatments; (C and D) Immunofluorescence staining results of HBMECs under different treatments; (E and F) Transwell analysis of HBMECs under different treatment; (G) Permeability changes of HBMECs under different treatment; (H) Pathway analysis of HBMECs under different treatments based on RNA sequencing.

(Date were presented as means ± standard deviations, ** indicates $P < 0.01$, *** indicates $P < 0.001$, **** indicates $P < 0.0001$.)

Supplementary Figure 8

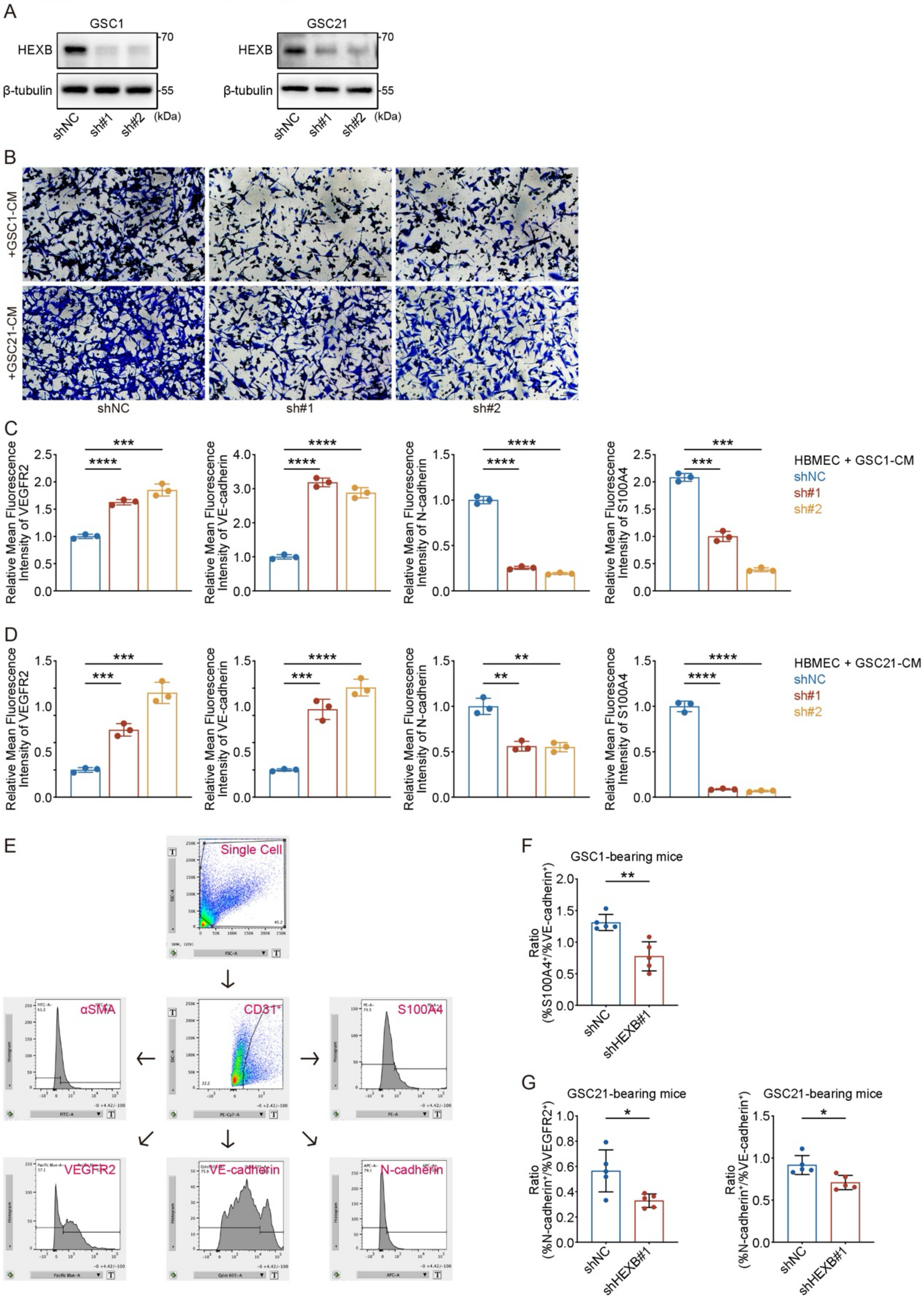

Figure S8. HEXB deficiency reduced the Endo-MT changes.

(A) Western blot results of gliomaspheres transfected with HEXB knockdown lentivirus (sh#1: shHEXB#1; sh#2: shHEXB#2); (B) Transwell analysis of HBMEC under different treatment (sh#1: shHEXB#1; sh#2: shHEXB#2); (C and D) Statistical analysis of immunofluorescence staining of HBMEC under different treatment (sh#1:

shHEXB#1; sh#2: shHEXB#2); (E) Representative flow-cytometry gating strategy for quantifying Endo-MT status of ECs in the tumors from SB-bearing C57BL6 mice; (F) Flow cytometry of $CD31^+$ cells in tumors from GSC1-bearing mice. (G) Flow cytometry of $CD31^+$ cells in tumors from GSC21-bearing mice.

(Date were presented as means ± standard deviations, * indicates $P \le 0.05$, ** indicates $P < 0.01$, *** indicates $P < 0.001$, **** indicates $P < 0.0001$.)

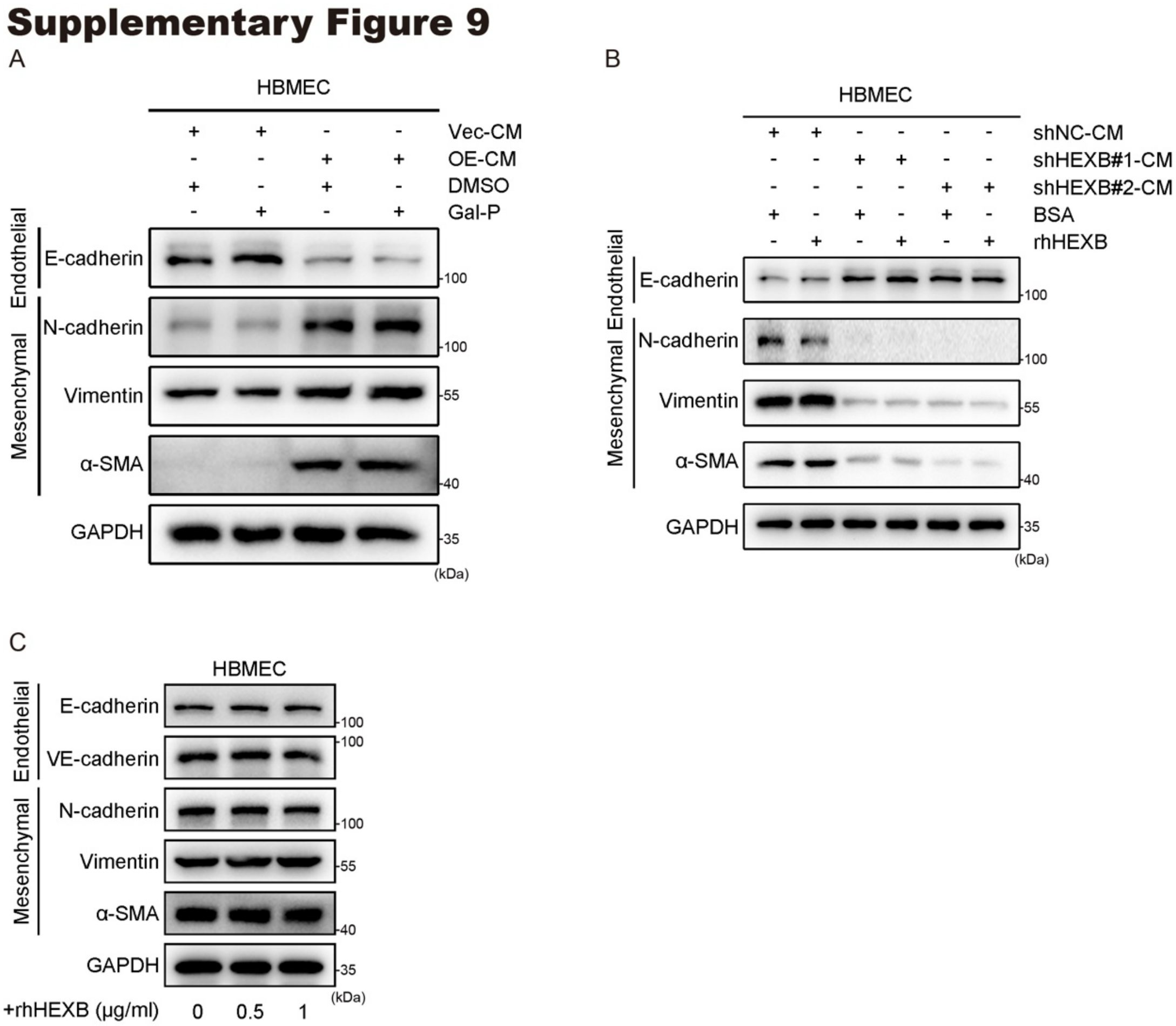

Figure S9. HEXB participated in Endo-MT in an indirect manner.

(A) Western blot results of HBMECs under different treatments with or without Gal-PUGNAC treatment; (B) Western blot results of HBMECs under different treatments with or without recombinant human HEXB protein treatment; (C) Western blot results of HBMECs with recombinant human HEXB protein treatment directly.

Supplementary Figure 10

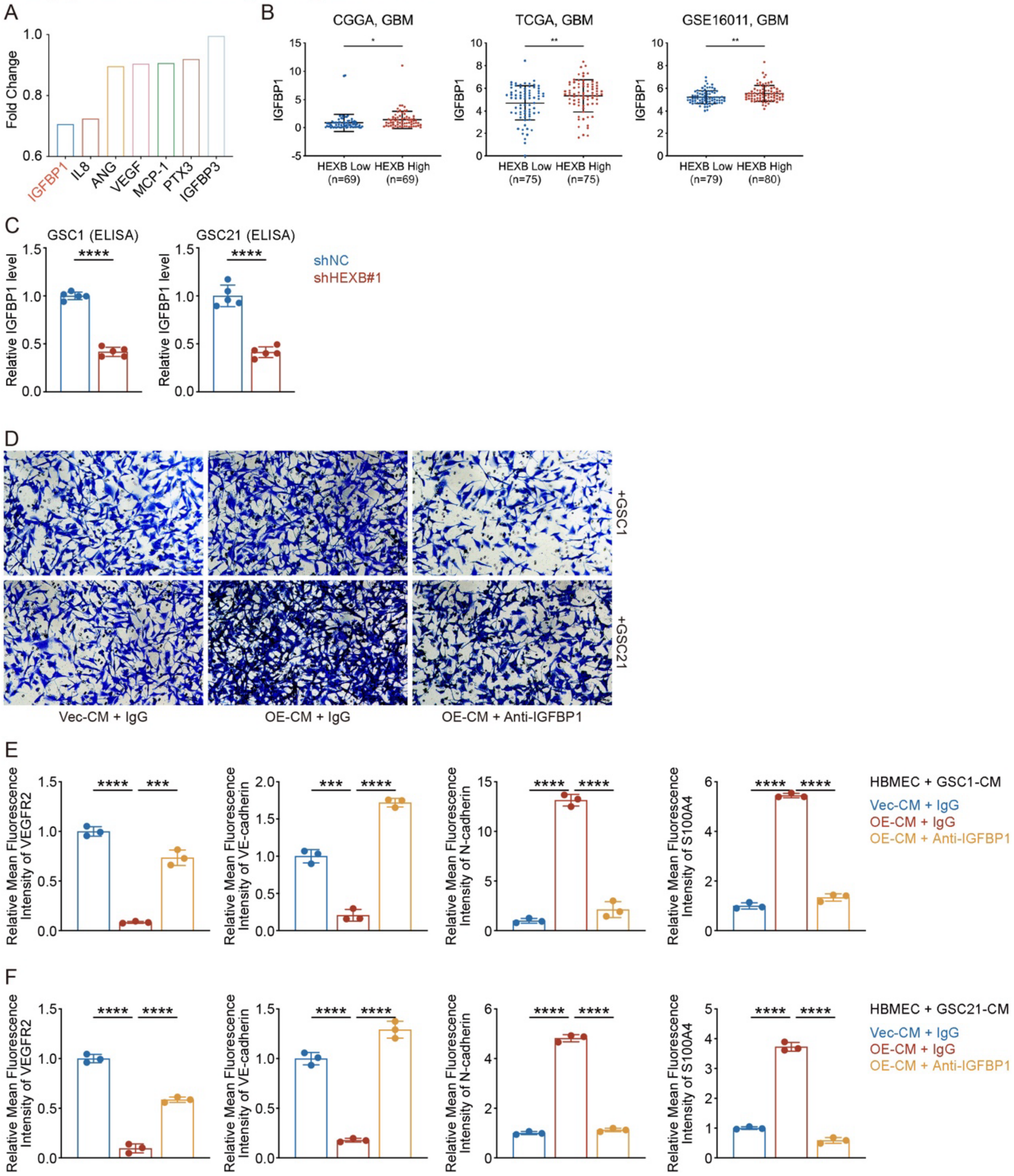

Figure S10. HEXB promoted Endo-MT changes through IGFBP1.

(A) Statistical analysis of angiogenesis-related array after HEXB knockdown; (B) The expression pattern of IGFBP1 in different group based on CGGA, TCGA, and GSE16011 GBM databases; (C) ELISA results of GSC1 and GSC21 under HEXB knockdown; (D) Transwell analysis of HBMEC under different treatment (Anti-IGFBP-1 neutralizing antibody was added into CM at a dose of 10 μg/ml); (E and F) Statistical analysis of immunofluorescence staining of HBMEC under different treatment (Anti-IGFBP-1 neutralizing antibody was added into CM at a dose of 10 μg/ml).

(Date were presented as means ± standard deviations, * indicates P ≤ 0.05, ** indicates P < 0.01, *** indicates P < 0.001, **** indicates P < 0.0001.)

Supplementary Figure 11

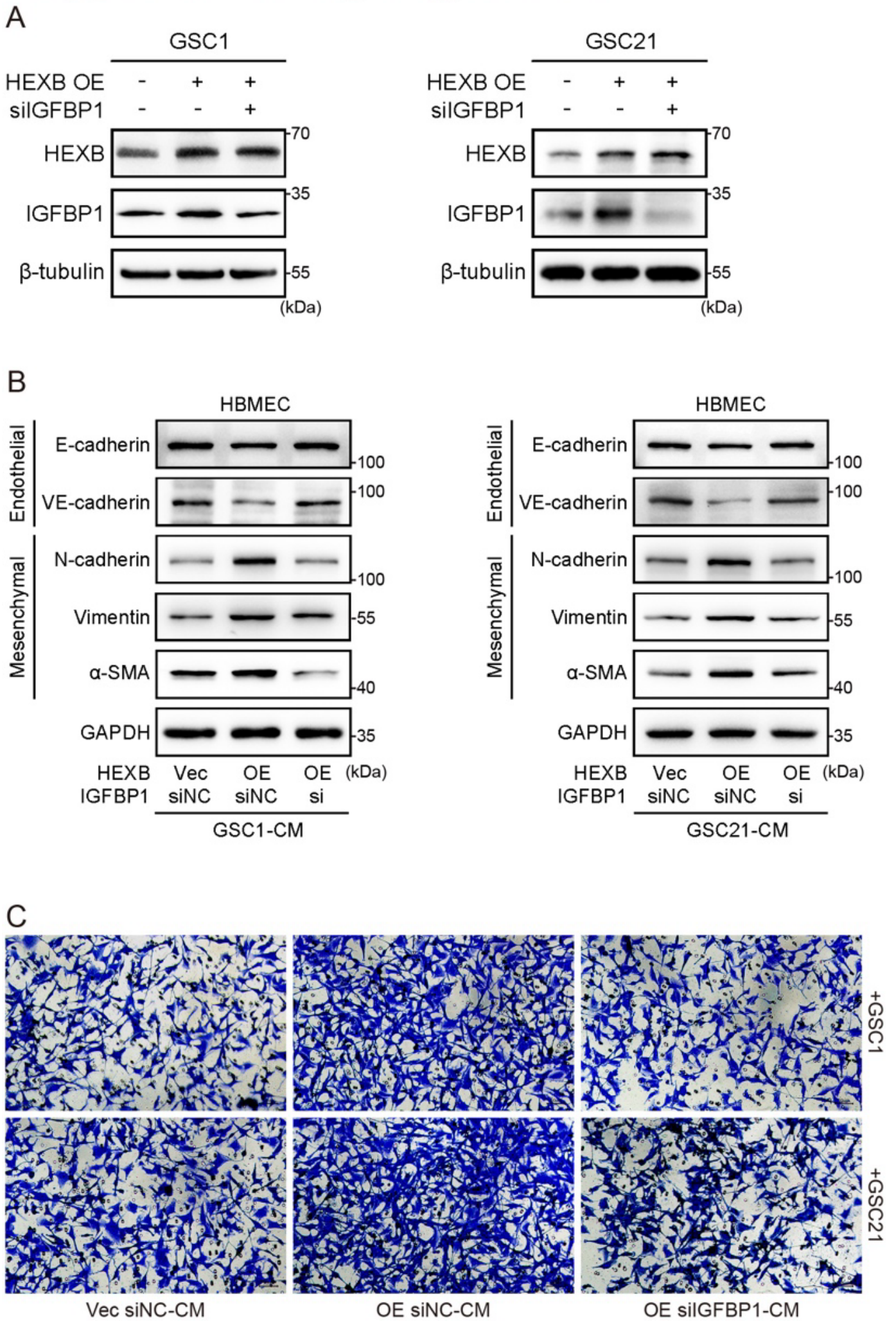

Figure S11. Blocking IGFBP1 reversed the promoting role of HEXB on Endo-MT status.

(A) Western blot results of GSC1 and GSC21 under different treatments; (B) Western blot results of HBMECs under different treatments; (C) Transwell analysis of HBMEC under different treatment.

Supplementary Figure 12

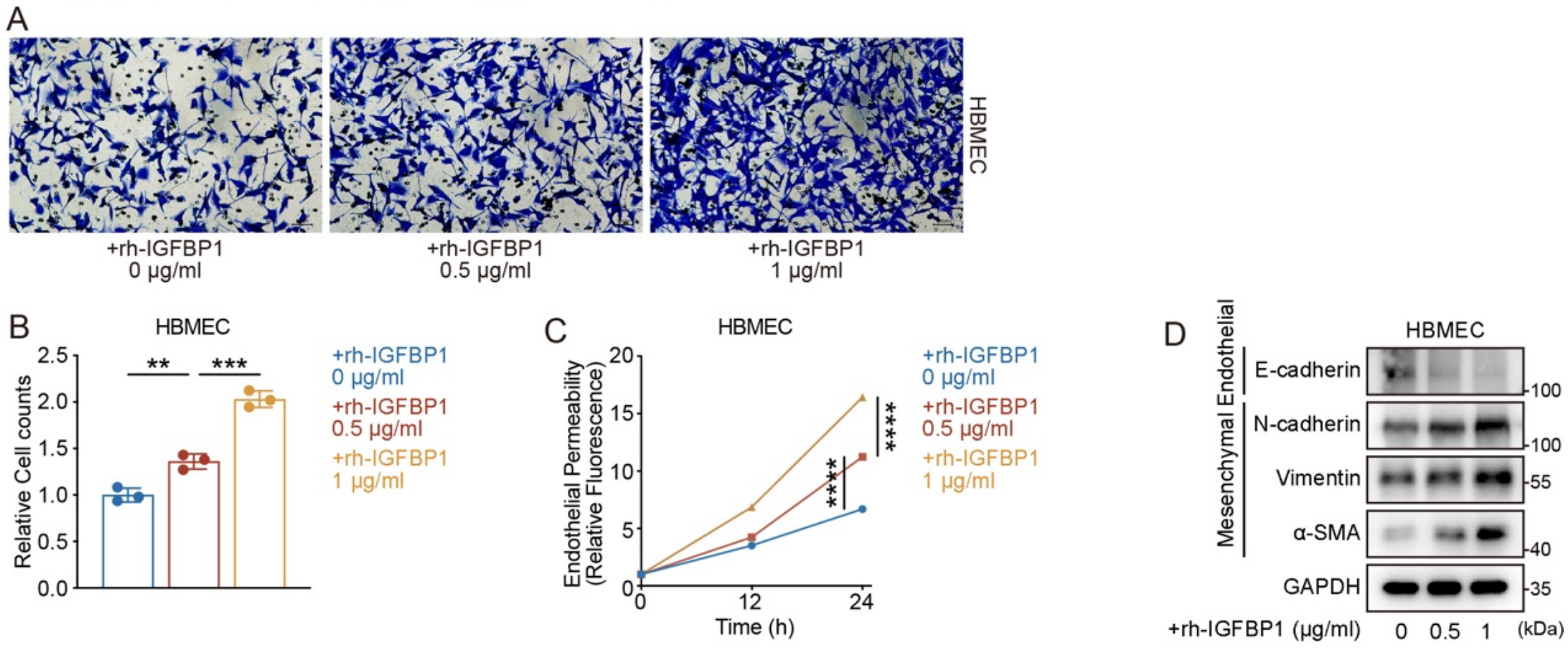

Figure S12. IGFBP1 treatment promoted Endo-MT status.

(A and B) Transwell analysis of HBMEC under different treatment (Recombinant IGFBP-1 protein was applied to HBMECs for 24 hours at different doses); (C) Permeability changes of HBMECs under different treatment (Recombinant IGFBP-1 protein was applied to HBMECs for 24 hours at different doses); (D) Western blot results of HBMECs under different treatments (Recombinant IGFBP-1 protein was applied to HBMECs for 24 hours at different doses).

(Date were presented as means ± standard deviations, ** indicates $P < 0.01$, *** indicates $P < 0.001$, **** indicates $P < 0.0001$.)

Supplementary Figure 13

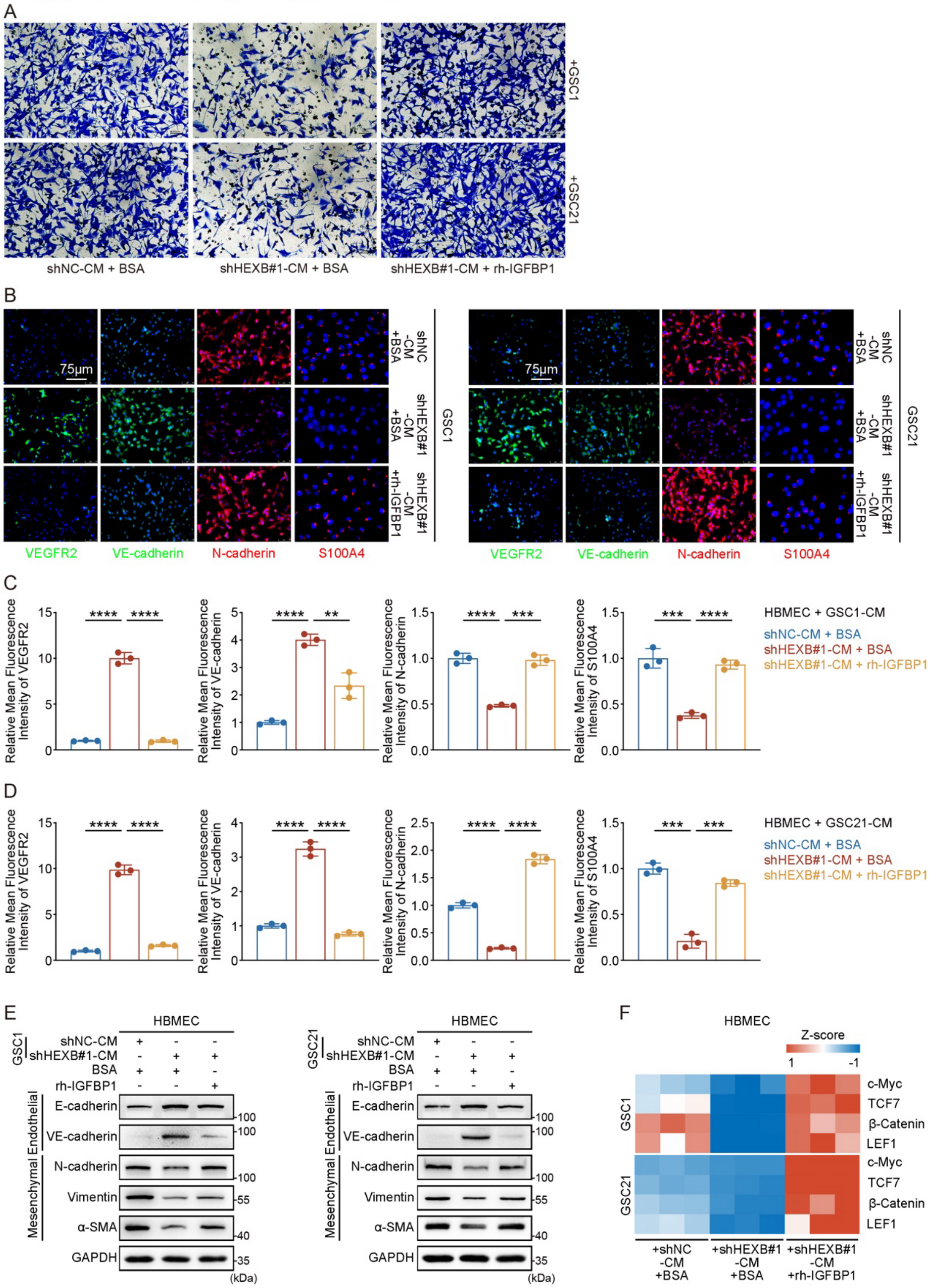

Figure S13. IGFBP1 treatment reversed the effect of HEXB knockdown on Endo-MT status.

(A) Transwell analysis of HBMEC under different treatment (Recombinant IGFBP-1 protein was added into CM at a dose of 1 μg/ml); (B-D) The analysis and represented immunofluorescence staining image of HBMECs under different treatments (Recombinant IGFBP-1 protein was added into CM at a dose of 1 μg/ml); (E) Western

blot results of HBMECs under different treatments (Recombinant IGFBP-1 protein was added into CM at a dose of 1 μg/ml); (F) PCR results of Wnt signaling markers in HBMECs under different treatments (Recombinant IGFBP-1 protein was added into CM at a dose of 1 μg/ml).

(Date were presented as means ± standard deviations, ** indicates P < 0.01, *** indicates P < 0.001, **** indicates P < 0.0001.)

Supplementary Figure 14

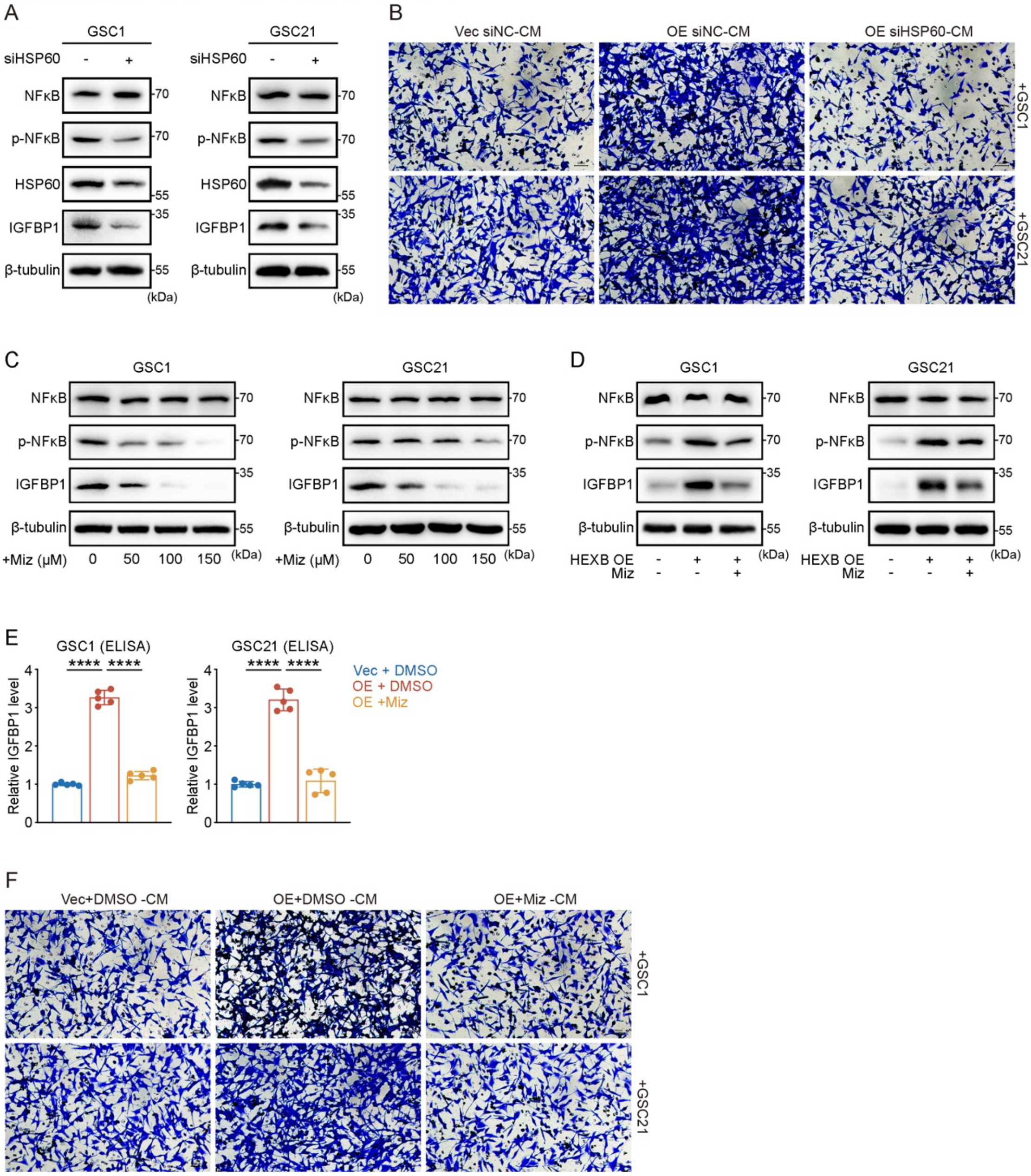

Figure S14. HSP60 was the mediator in HEXB/IGFBP1/Endo-MT.

(A) Western blot results of GSC1 and GSC21 under HSP60 knockdown; (B) Transwell analysis of HBMEC under different treatment; (C) Western blot results of GSC1 and GSC21 under HSP60 inhibition (Miz was applied to GSCs for 24 hours at

different doses); (D) Western blot results of GSC1 and GSC21 under different treatment (Miz was applied to indicated GSCs at a dose of 100 μM for 24 hours prior to CM collection); (E) ELISA results of GSC1 and GSC21 under different treatment (Miz was applied to indicated GSCs at a dose of 100 μM for 24 hours prior to CM collection); (F) Transwell analysis of HBMEC under different treatment (Miz was applied to indicated GSCs at a dose of 100 μM for 24 hours prior to CM collection).
(Date were presented as means ± standard deviations, **** indicates P < 0.0001.)

Supplementary Figure 15

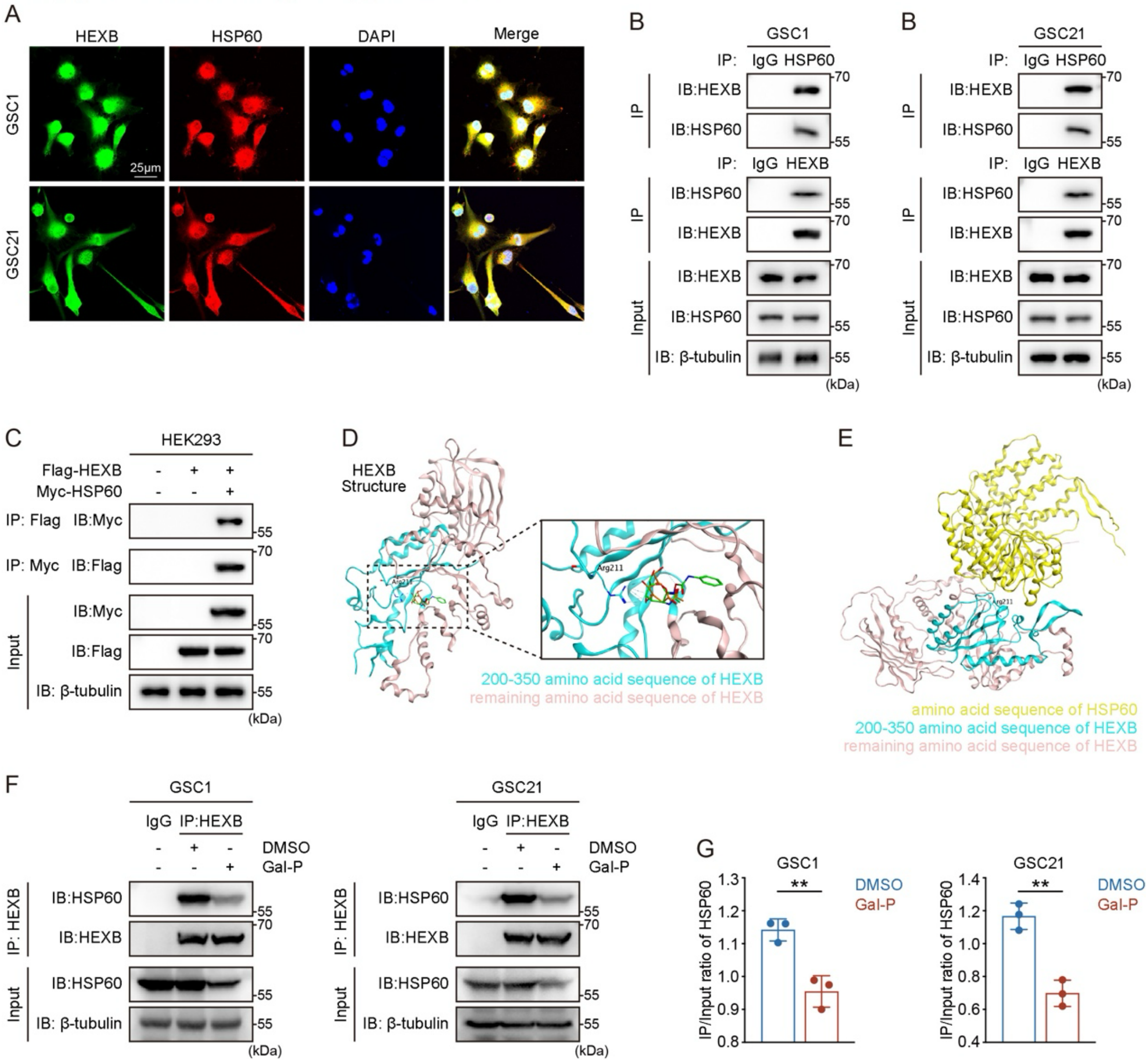

Figure S15. HEXB bind with HSP60.

(A) Dual immunofluorescence of HEXB and HSP60 in GSC1 and GSC21; (B) The interaction between HEXB and HSP60 by co-immunoprecipitation in GSC1 and GSC21; (C) The interaction between FLAG-tagged HEXB and Myc-tagged HSP60 by co-immunoprecipitation in HEK293 cells; (D) The molecular docking of HEXB and Gal-P (The cyan part represents the 200-350 amino acid sequence structure while the pink part shows the amino acids of other sequences in HEXB); (E) The molecular docking of HEXB and HSP60 (Yellow represents HSP60, cyan represents the 200-350 amino acid sequence structure of HEXB, and pink denotes the remaining

sequence of the HEXB protein); (F and G) Gal-P treatment inhibited the interaction between HEXB and HSP60.
(Date were presented as means ± standard deviations, ** indicates P < 0.01.)

Supplementary Figure 16

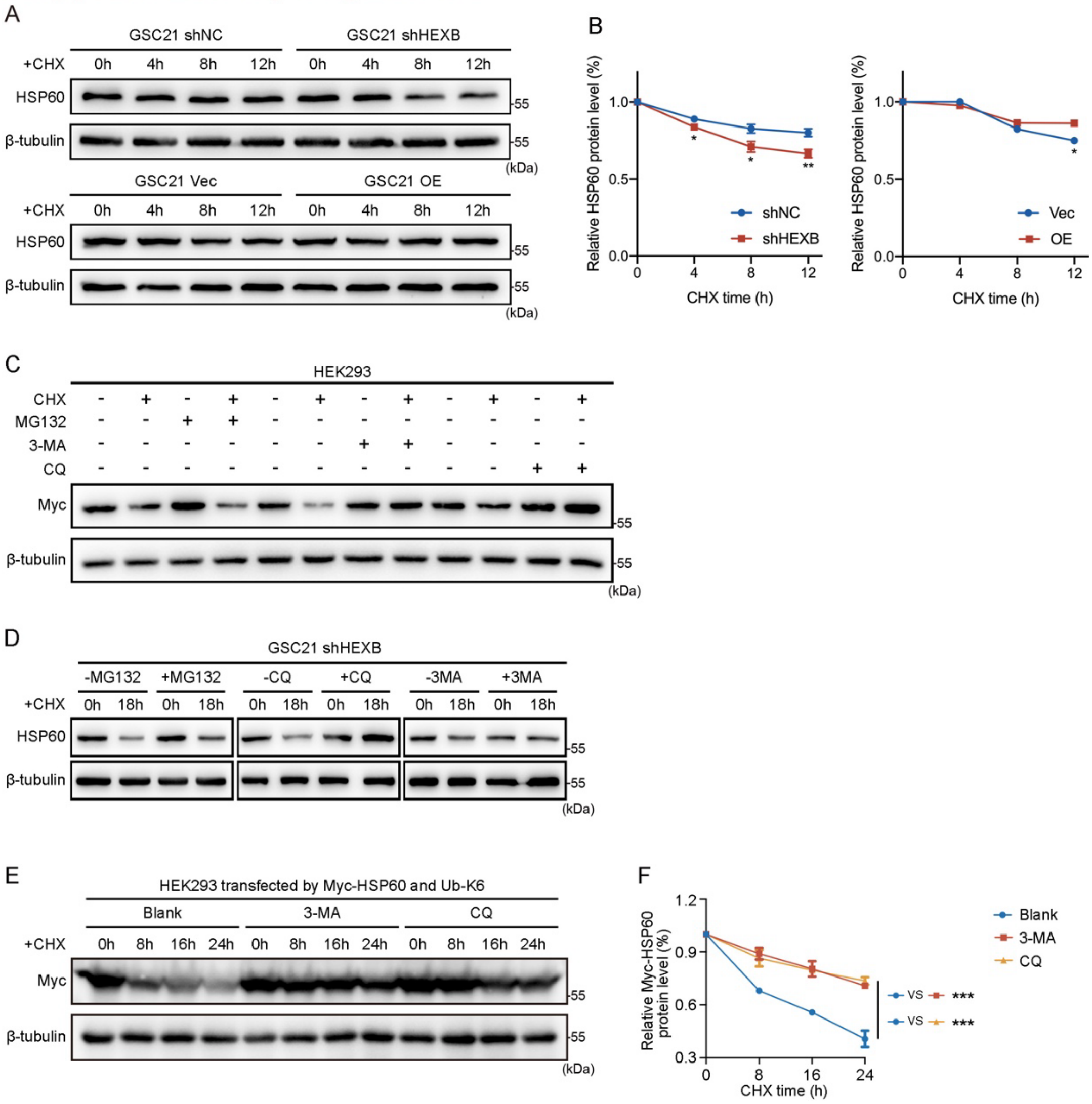

Figure S16. HEXB inhibited HSP60 degradation.
(A) Representative western blot images of time-dependent HSP60 expression in GSC21 cells under different treatments (CHX was applied to indicated cells at a dose of 100 μg/ml for different hours); (B) The degradation curve of time-dependent HSP60 expression in GSC21 cells under different treatments; (C) Western blot results of HEK293 cells under different treatments (CHX was applied to cells at a dose of 100 μg/ml for 18 hours; MG132 was applied to cells at a dose of 10 μM for 6 hours prior to CHX addition; 3-MA was applied to cells at a dose of 5 mM for 6 hours prior to CHX addition; CQ was applied to cells at a dose of 20 μM for 6 hours prior to CHX addition); (D) Western blot results of GSC21 shHEXB cells under different treatments (CHX was applied to cells at a dose of 100 μg/ml for 18 hours; MG132

was applied to cells at a dose of 10 μM for 6 hours prior to CHX addition; 3-MA was applied to cells at a dose of 5 mM for 6 hours prior to CHX addition; CQ was applied to cells at a dose of 20 μM for 6 hours prior to CHX addition); (E) Representative western blot images of time-dependent Myc-tagged HSP60 expression in HEK293 cells under different treatments (CHX was applied to cells at a dose of 100 μg/ml for different hours; MG132 was applied to cells at a dose of 10 μM for 6 hours prior to CHX addition; 3-MA was applied to cells at a dose of 5 mM for 6 hours prior to CHX addition; CQ was applied to cells at a dose of 20 μM for 6 hours prior to CHX addition); (F) The degradation curve of time-dependent Myc-tagged HSP60 expression in HEK293 cells under different treatments.

(Date were presented as means ± standard deviations, * indicates P ≤ 0.05, ** indicates P < 0.01, *** indicates P < 0.001.)

Supplementary Figure 17

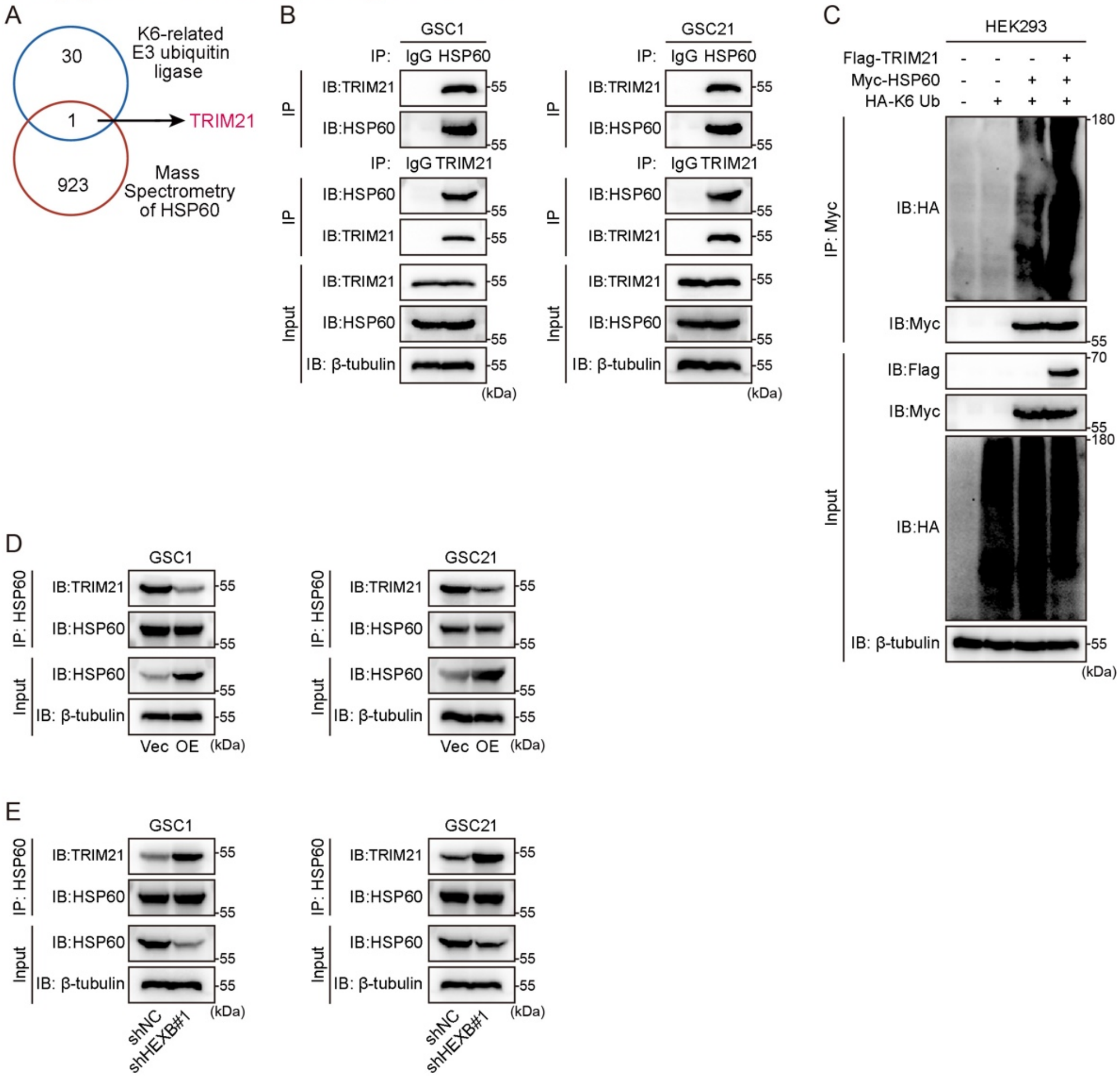

Figure S17. TRIM21 interacted with HSP60 and promoted its K6-linked ubiquitination.

(A) Venn diagram illustrating the screening strategy for the specific E3 ubiquitin ligase of HSP60; (B) Co-IP assay demonstrating the endogenous interaction between TRIM21 and HSP60 in GSCs; (C) TRIM21 promoted the K6-linked ubiquitination of

HSP60; (D) Co-IP analysis demonstrating that HEXB inhibited the interaction between TRIM21 and HSP60 in GSCs; (E) Co-IP analysis demonstrating that HEXB knockdown promoted the interaction between TRIM21 and HSP60 in GSCs.

Supplementary Figure 18

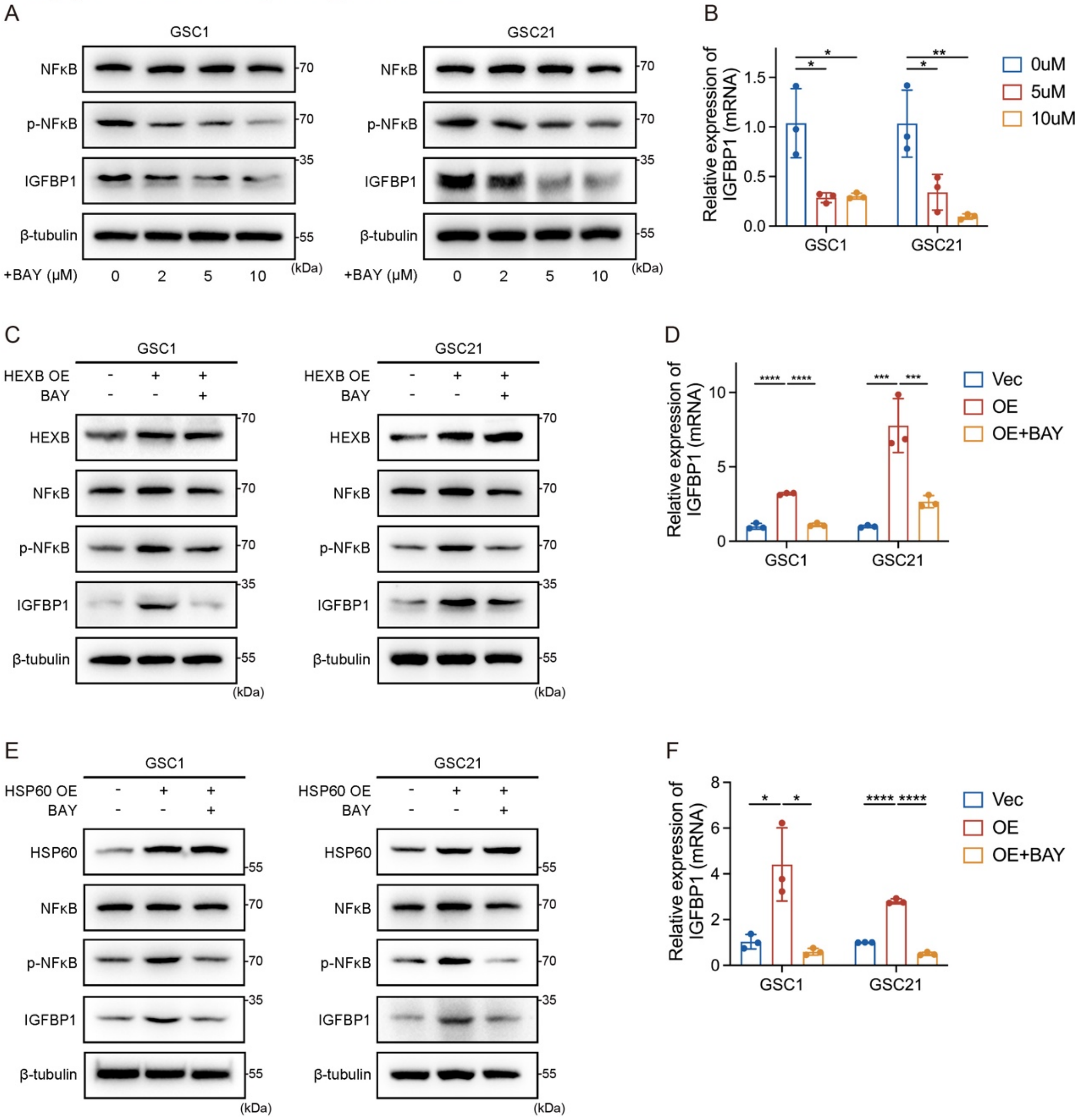

Figure S18. The HEXB-HSP60 complex regulated IGFBP1 transcription via activating NFκB signaling.

(A) Western blot results of gliomaspheres under NFκB inhibitor BAY11-7082 treatments (BAY11-7082 was applied to GSCs for 24 hours at different doses); (B) PCR results of gliomaspheres under NFκB inhibitor BAY11-7082 treatments (BAY11-7082 was applied to GSCs for 24 hours at different doses); (C) Western blot results of gliomaspheres under different treatments (BAY11-7082 was applied to indicated GSCs at a dose of 10 μM for 24 hours); (D) PCR results of gliomaspheres under different treatments (BAY11-7082 was applied to indicated GSCs at a dose of 10 μM for 24 hours); (E) Western blot results of gliomaspheres under different

treatments (BAY11-7082 was applied to indicated GSCs at a dose of 10 μM for 24 hours); (F) PCR results of gliomaspheres under different treatments (BAY11-7082 was applied to indicated GSCs at a dose of 10 μM for 24 hours).
(Date were presented as means ± standard deviations, * indicates P ≤ 0.05, ** indicates P < 0.01, *** indicates P < 0.001, **** indicates P < 0.0001.)

Supplementary Figure 19

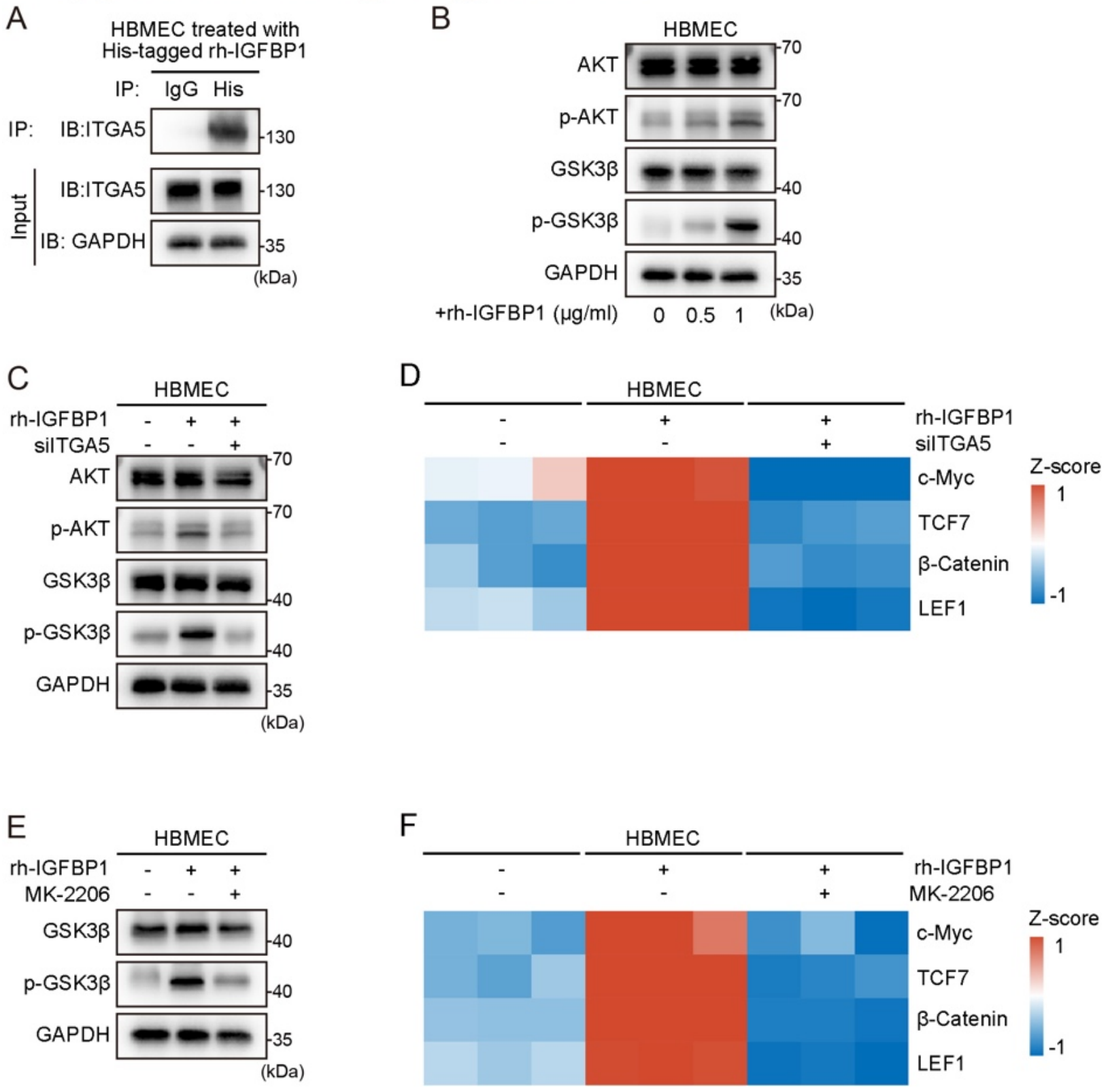

Figure S19. HEXB/IGFBP1 activated Wnt signaling in ECs through ITGA5/AKT/GSK3β.

(A) Co-immunoprecipitation results of HBMECs treated with His-tagged recombinant IGFBP1 protein (Recombinant IGFBP-1 protein was applied to HBMECs at a dose of 1 μg/ml for 24 hours); (B) Western blot results of indicated proteins in HBMECs under different dose of recombinant IGFBP1 protein (Recombinant IGFBP-1 protein was applied to HBMECs for 24 hours at different doses); (C) Western blot results of indicated proteins in HBMECs under different treatments; (D) PCR results of HBMECs under different treatments; (E) Western blot results of indicated proteins in HBMECs under different treatments (Recombinant IGFBP-1 protein was applied to HBMECs at a dose of 1 μg/ml for 24 hours; MK-2206 was applied to HBMECs at a dose of 1 μM for 24 hours); (F) PCR results of HBMECs under different treatments (Recombinant IGFBP-1 protein was applied to HBMECs at a dose of 1 μg/ml for 24 hours; MK-2206 was applied to HBMECs at a dose of 1 μM for 24 hours).

Supplementary Figure 20

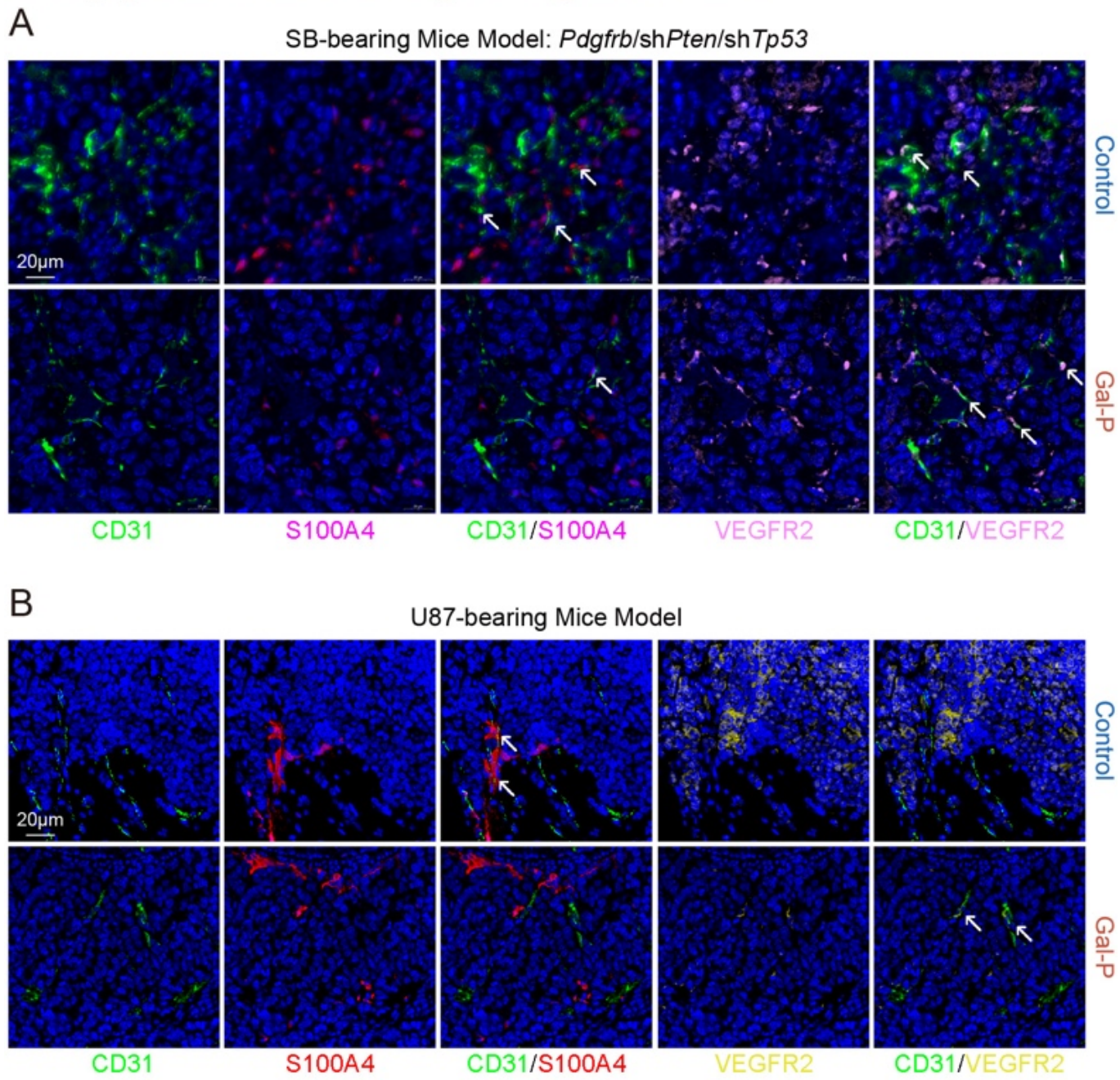

Figure S20. HEXB inhibition reversed Endo-MT status *in vivo*.

(A) The representative images of three-color immunofluorescence staining of tumors from SB-bearing C57BL6 mice under different treatment; (B) The representative images of three-color immunofluorescence staining of tumors from U87-bearing BALB/c nude mice under different treatment.

Supplementary Figure 21

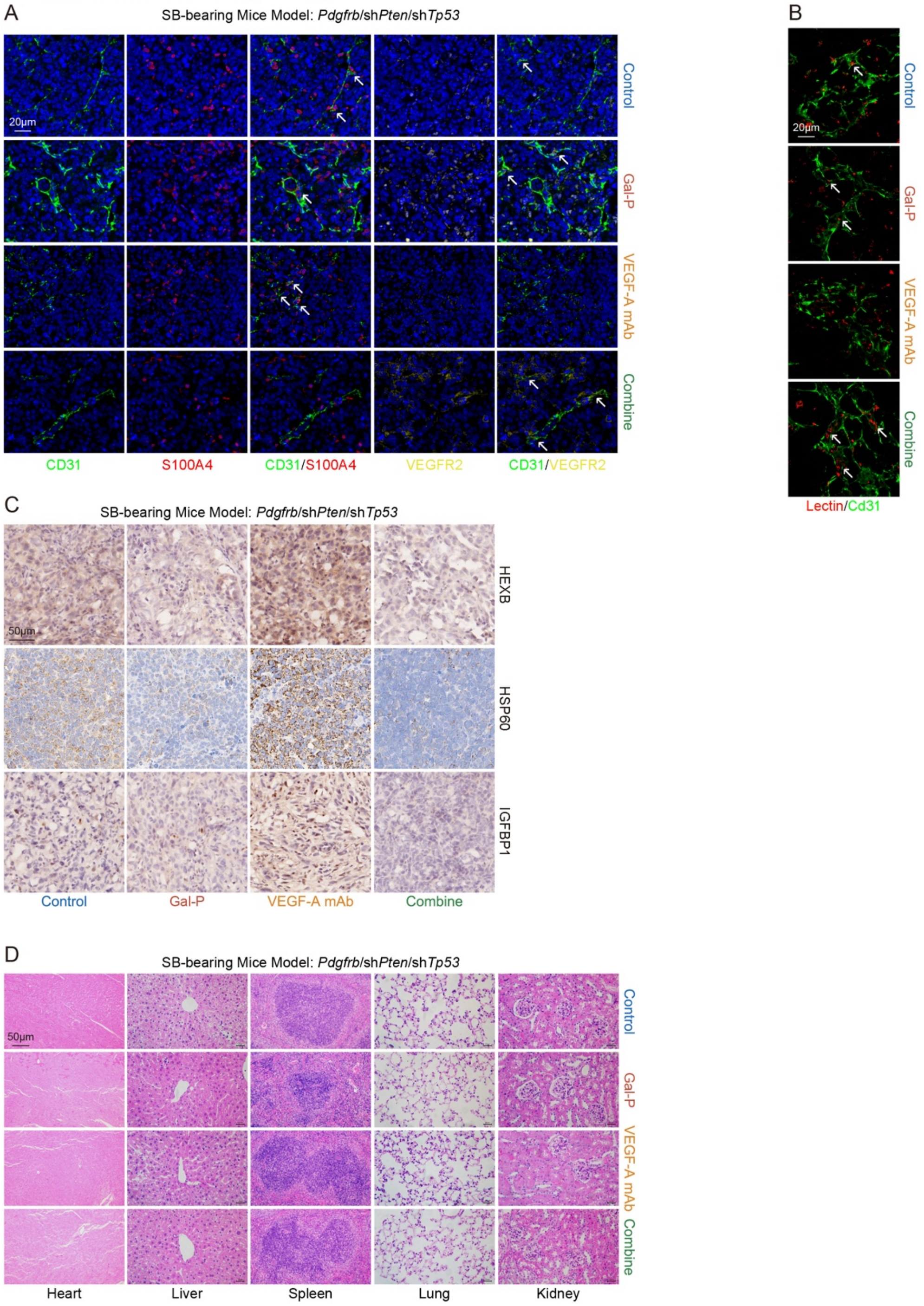

Figure S21. HEXB inhibition promoted VEGF-A antibody therapeutic efficacy *in vivo*.

(A) The representative images of three-color immunofluorescence staining of tumors from SB-bearing C57BL6 mice under different treatment; (B) The representative images of lectin-positive vessels in total vessels in tumors from SB-bearing C57BL6 mice under different treatment; (C) The representative images of HEXB, HSP60 and IGFBP1 staining of tumors from SB-bearing C57BL6 mice under different treatment; (D) The representative H&E staining image of heart, liver, spleen, lung and kidney under different treatment.

Supplementary Figure 22

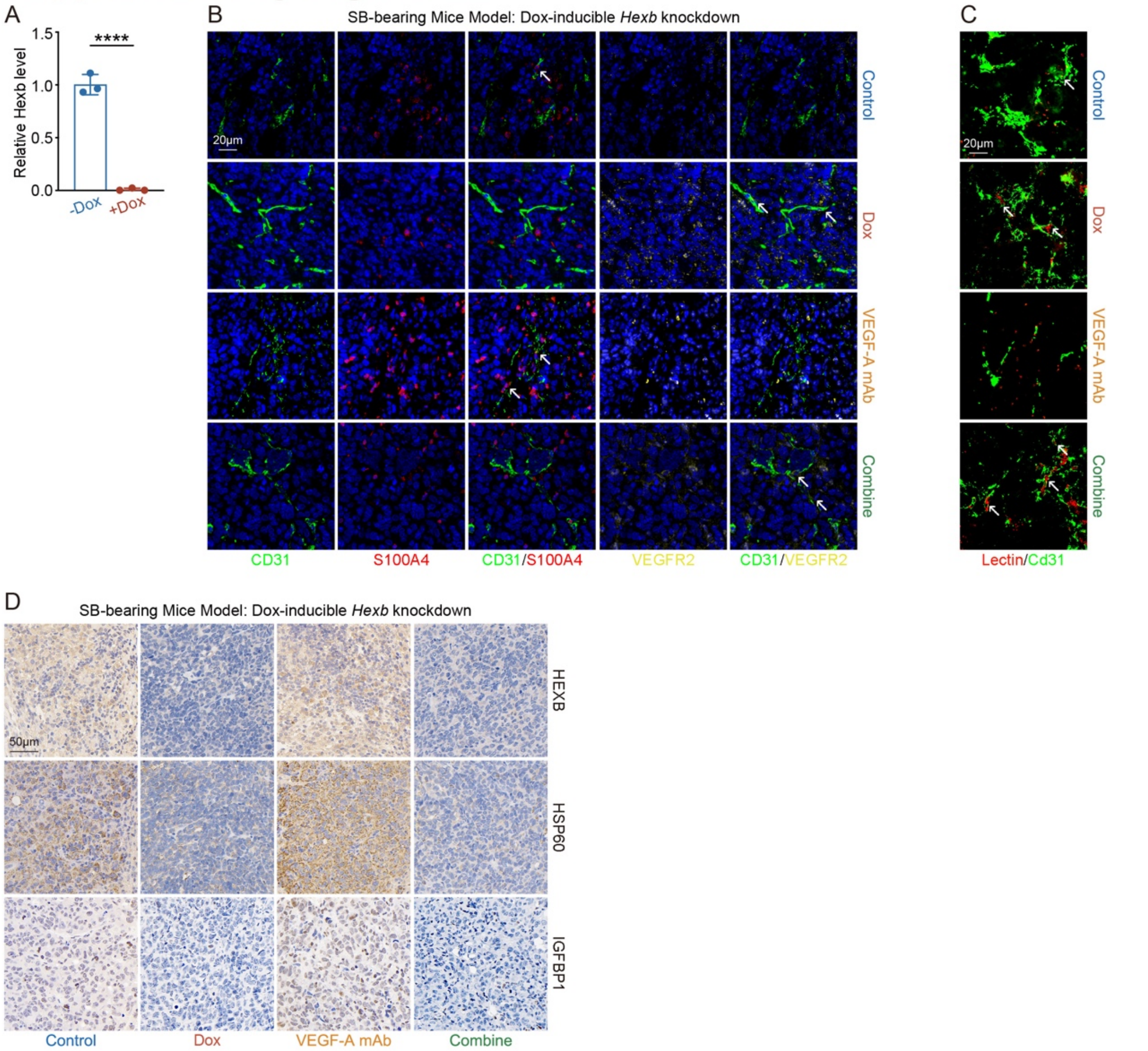

Figure S22. Genetic ablation of *Hexb* promoted VEGF-A antibody therapeutic efficacy *in vivo*.

(A) Detection of *Hexb* knockdown efficiency following Dox treatment; (B) The representative images of three-color immunofluorescence staining of tumors from SB-bearing C57BL6 mice under different treatment; (C) The representative images of lectin-positive vessels in total vessels in tumors from SB-bearing C57BL6 mice under different treatment; (D) The representative images of HEXB, HSP60 and IGFBP1 staining of tumors from SB-bearing C57BL6 mice under different treatment.

(Date were presented as means ± standard deviations, **** indicates $P < 0.0001$.)
